# Past and ongoing adaptation of human cytomegalovirus to its host

**DOI:** 10.1371/journal.ppat.1008476

**Published:** 2020-05-08

**Authors:** Alessandra Mozzi, Matteo Biolatti, Rachele Cagliani, Diego Forni, Valentina Dell'Oste, Chiara Pontremoli, Chiara Vantaggiato, Uberto Pozzoli, Mario Clerici, Santo Landolfo, Manuela Sironi

**Affiliations:** 1 Scientific Institute, IRCCS E. MEDEA, Bioinformatics, Bosisio Parini, Italy; 2 Laboratory of Pathogenesis of Viral Infections, Department of Public Health and Pediatric Sciences, University of Turin, Turin, Italy; 3 Scientific Institute, IRCCS E. MEDEA, Laboratory of Molecular Biology, Bosisio Parini, Italy; 4 Department of Physiopathology and Transplantation, University of Milan, Milan, Italy; 5 Don C. Gnocchi Foundation ONLUS, IRCCS, Milan, Italy; Emory University Vaccine Center, UNITED STATES

## Abstract

Cytomegaloviruses (order *Herpesvirales*) display remarkable species-specificity as a result of long-term co-evolution with their mammalian hosts. Human cytomegalovirus (HCMV) is exquisitely adapted to our species and displays high genetic diversity. We leveraged information on inter-species divergence of primate-infecting cytomegaloviruses and intra-species diversity of clinical isolates to provide a genome-wide picture of HCMV adaptation across different time-frames. During adaptation to the human host, core viral genes were commonly targeted by positive selection. Functional characterization of adaptive mutations in the primase gene (*UL70*) indicated that selection favored amino acid replacements that decrease viral replication in human fibroblasts, suggesting evolution towards viral temperance. HCMV intra-species diversity was largely governed by immune system-driven selective pressure, with several adaptive variants located in antigenic domains. A significant excess of positively selected sites was also detected in the signal peptides (SPs) of viral proteins, indicating that, although they are removed from mature proteins, SPs can contribute to viral adaptation. Functional characterization of one of these SPs indicated that adaptive variants modulate the timing of cleavage by the signal peptidase and the dynamics of glycoprotein intracellular trafficking. We thus used evolutionary information to generate experimentally-testable hypotheses on the functional effect of HCMV genetic diversity and we define modulators of viral phenotypes.

## Author summary

Human cytomegalovirus (HCMV), which represents the most common infectious cause of birth defects, is perfectly adapted to infect humans. We performed a two-tier analysis of HCMV evolution, by describing selective events that occurred during HCMV adaptation to our species and by identifying more recently emerged adaptive variants in clinical isolates. We show that distinct viral genes were targeted by natural selection over different time frames and we generate a catalog of adaptive variants that represent candidate determinants of viral phenotypic variation. As a proof of concept, we show that adaptive changes in the viral primase modulate viral growth in vitro and that selected variants in the UL144 signal peptide affect glycoprotein intracellular trafficking.

## Introduction

Cytomegaloviruses (CMVs, family *Herpesviridae*) infect many mammals, including humans and other primates [1]. Human cytomegalovirus (HCMV) infection is very common, with worldwide seroprevalence ranging from 56% to 94% [2]. Whereas HCMV is generally asymptomatic in healthy adults, the virus is an important opportunistic pathogen among immunocompromised individuals such as AIDS patients and transplant recipients [3]. HCMV is also the most common infectious cause of birth defects [4, 5]. Like other herpesviruses, HCMV establishes a persistent infection through latency and recent data suggest that the virus has long-term clinical consequences, especially in the elderly [6].

HCMV possesses the largest genome (~235,000 bp) among human herpesviruses and, more generally, among viruses known to infect humans [7]. Protein-coding genes occupy the great majority of the HCMV genome and are generally divided into core genes, which are shared by all herpesviruses, and non-core or sub-core genes [7–10]. These are specific to herpesvirus genera or even to CMV species and, in contrast to core genes, are often dispensable for viral growth in cell culture [9, 11]. Nonetheless, several non-core proteins play important roles during infection *in vivo*, indicating that most genes contribute to the success of CMVs in natural hosts [1, 9, 12].

CMVs display remarkable species-specificity, which results from long-term co-evolution with and adaptation to their mammalian hosts [13, 14]. Comparison of the HCMV genome with those of non human primate-infecting CMVs indicated that duplication and gene copy number variation of non-core genes were common during CMV evolution and probably contributed to host adaptation [1, 10]. However, coding gene sequence evolution and episodes of positive selection are also known to frequently shape host-pathogen interactions and to contribute to host switches [15].

HCMV genetic diversity is higher than that observed for other human-infecting herpesviruses [16] and mixed infections caused by genetically distant strains are also common [17]. Thus, the within-host HCMV genetic diversity can be very high, suggesting that HCMV populations are the target of strong selective pressures exerted by the host and by competing viral strains. Herein, we leveraged information on inter-species divergence in primate-infecting CMVs and intra-species diversity in HCMV clinical isolates to provide a global picture of HCMV evolution across different time frames.

## Results

### Evolution of primate CMV coding genes

**Fig 1:**
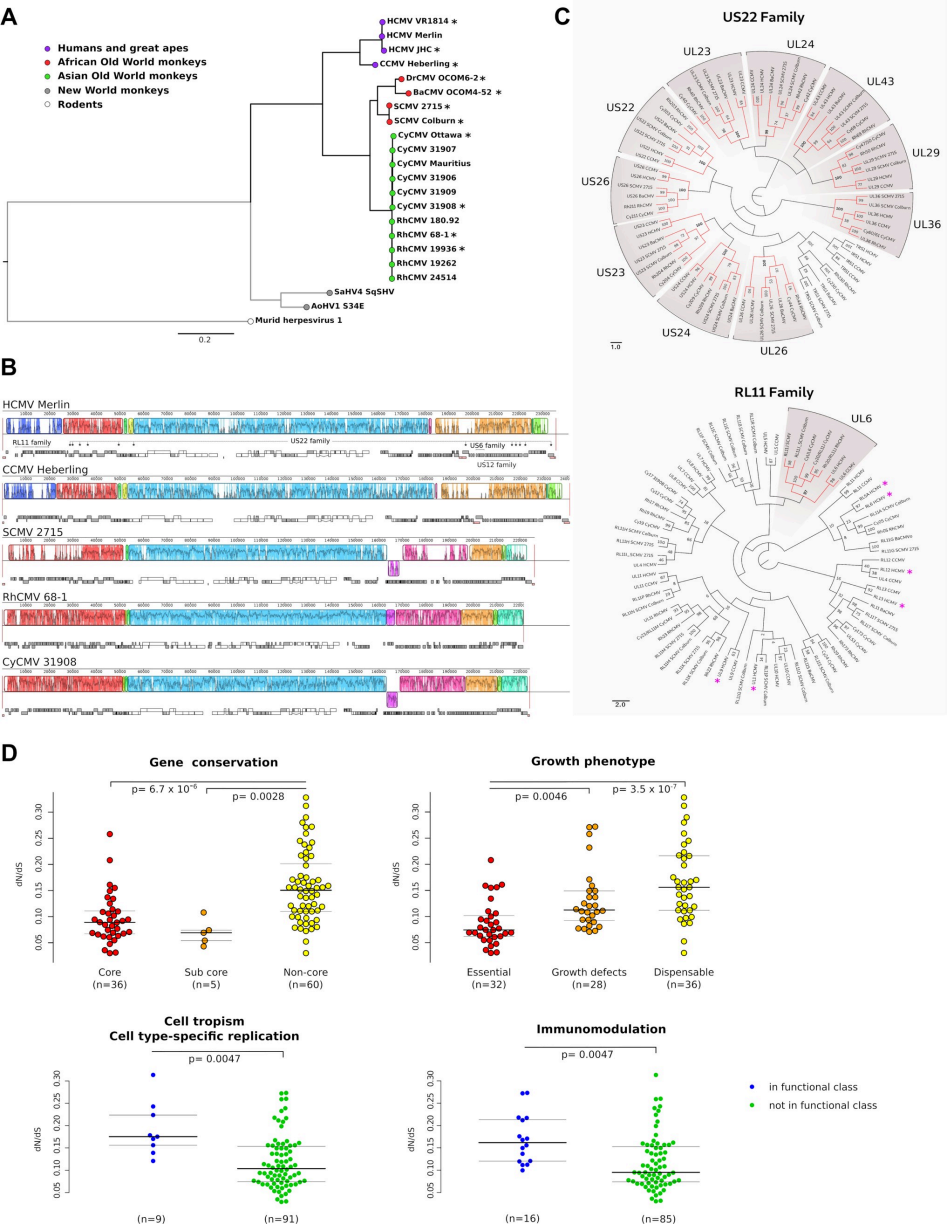
Evolution of catarrhini-infecting CMVs. **(A)** A maximum-likelihood tree of the full-length amino acid sequence of alkaline nuclease (encoded by the core gene *UL98*) is drawn to exemplify phylogenetic relationships among primate CMVs (CCMV, Chimpanzee cytomegalovirus; DrCMV, Drill monkey cytomegalovirus; BaCMV, Chacma baboon cytomegalovirus; SCMV, Simian cytomegalovirus; CyCMV, Cynomolgus macaque cytomegalovirus; RhCMV, Rhesus macaque cytomegalovirus; SaHV4, Squirrel monkey cytomegalovirus; AoHV1, Owl monkey cytomegalovirus). Murine CMV (murid herpesvirus 1) was used as the outgroup and the tree was constructed using RAxML (version 8.2.12) [19]. Asterisks denote viruses that were included in the analysis of selective patterns of catarrhini-infecting CMVs. **(B)** Whole-genome alignment of four representative primate CMVs obtained with progressive MAUVE. Each genome is laid out in a horizontal track, with annotated coding regions shown as boxes (white: core genes, gray: non-core genes); repetitive elements are shown as orange boxes. A colored similarity plot generated by progressive MAUVE is also shown: each colored block delimits a genome region that aligns to part of another genome (presumably homologous and free from internal rearrangements) and thus represents a locally collinear block. A similarity profile is plotted within blocks, with its height proportional to the average level of conservation in that region. White areas correspond to regions that could not be aligned. When the similarity plot points downward it indicates an alignment to the reverse strand of the genome. The location of genes belonging to the *US22*, *US12*, *RL11*, and *US6* families is shown. **(C)** Phylogenetic relationships for large gene families. The protein sequences of family homologs were searched for as described in the Materials and Methods. Phylogenetic trees were constructed using RAxML with 1000 bootstrap replicates (reported at nodes). Orthologous gene groups, shown in red on the tree and denoted by the gray shading, were inferred on the basis of the tree topology and of bootstrap values > 90. Magenta asterisks denote genes that are frequently deleted/mutated in clinical isolates [16]. **(D)** Analysis of selective patterns. The dN/dS parameter is compared among genes showing different levels of sequence conservation and distinct growth phenotypes (upper panels). Growth phenotypes in human fibroblasts were obtained from a previous work [11] that merged data from two systematic analyses of gene disruption [18, 20]. Statistical significance was assessed by Kruskal-Wallis tests followed by Nemenyi tests as post-hocs (reported in the figure). In the lower panels, genes are grouped based on function. Functional categories were derived from a previous annotation effort that combined multiple information sources [11]. p values derive from Wilcoxon Rank-Sum tests with FDR correction.

We first aimed to explore the selective patterns of primate CMV coding genes. Because high sequence diversity can affect evolutionary inference, viruses that infect New World primates were excluded from these analyses. We thus analyzed representative, complete genomes of CMVs that infect great apes, Old World African monkeys, and Old world Asian monkeys (Fig 1A).

In line with previous observations [1, 9, 10], a whole-genome alignment revealed a large central collinear block, which encompasses the majority of core genes (Fig 1B). Partially due to the presence of gene families, regions flanking core genes are known to be dynamic in terms of gene content [1, 9, 10]. We thus applied a phylogenetic approach to explore gene orthology among members of the largest families (*US22*, *US12*, *RL11*, and *US6*). For the *US22* and *US12* families, one-to-one orthology could be inferred for most genes (Fig 1C and S1 Fig). Conversely, *RL11* and *US6* family members showed murky relationships, most likely due to duplication events that occurred at different time-points during primate CMV evolution (Fig 1C and S1 Fig). Several genes in these families were shown to be dispensable for HCMV growth and to be commonly disrupted in clinical isolates [16, 18] (Fig 1C and S1 Fig).

Analysis of selective patterns was thus performed for all coding genes with reliable one-to-one orthologs in 11 genomes selected to be representative of catarrhini-infecting CMVs (Fig 1A and S1 Table). Gene sequences were rigorously filtered to ensure high quality alignments (see Methods) and genes with short alignments were discarded (S2 Table*)*. Overall, we calculated the average non-synonymous substitution/synonymous substitution rate (dN/dS, also referred to as ω) for 101 genes. All genes had dN/dS values much lower than 1, indicating that purifying selection is the major force acting on CMV coding regions. Comparison among core, sub-core (conserved in *Betaherpesviridae* and *Gammaherpesviridae*), and non-core genes (specific to CMVs) indicated that these latter have significantly higher average dN/dS (Kruskal-Wallis rank sum test, p = 5.577*10–7, Nemenyi post-hoc tests are reported in Fig 1D). Consistently, essential genes showed greater evolutionary constraint than dispensable genes or genes that cause growth defects when deleted/mutated, (Kruskal-Wallis rank sum test, p *=* 6.6488*10–7, Nemenyi post-hoc tests are reported in Fig 1D). This observation should however be taken with caution, as growth phenotypes were determined for a cell culture-adapted HCMV strain [11] and do not necessarily correspond to phenotypes that would be observed in clinical isolates or other primate CMVs.

Among functional classes, genes involved in immunomodulation and cell-specific tropism displayed higher dN/dS than genes that have not been reported to participate in these processes (Fig 1D). Significant differences in evolutionary rates were also found among proteins that localize to the cell membrane or in the cell nucleus (S2 Fig). In agreement with this latter finding, genes that participate to DNA replication had lower dN/dS than genes that are not known to be involved in this process, although the difference was not significant (S2 Fig); the failure to reach statistical significance may be due to the small number of genes included in this functional class. In general, the statistical power to detect differences is low for functional classes/compartments with few annotated genes. Moreover, in these cases, the unbalanced sample sizes complicate the interpretation of the differences.

### Adaptive evolution in the HCMV lineage

We next assessed whether adaptation to the human host drove the evolution of specific coding genes in HCMV. We thus considered a phylogeny of primate CMVs that included all fully sequenced catarrhini-infecting CMVs (Fig 1A) and 25 HCMV clinical isolates (S1 Table). We applied a branch-site test [21] to detect positive selection that occurred on the HCMV branch (S2 and S3 Tables). In this test, the branches of the tree are divided a priori into foreground (here, the branch leading to all HCMV isolates) and background lineages (all other branches), and models that allow or disallow positive selection on the foreground lineage(s) are compared. The branch-site test can thus detect lineage-specific selected genes and sites (episodic positive selection).

After FDR (false discovery rate) correction, the test detected 34 positively selected genes (S3 Table). Despite their higher constraint during evolution in catarrhini (Fig 1D), core genes were more frequently targeted by positive selection (fraction selected = 41.7%) than non-core plus sub-core genes (fraction selected = 29.2%). Identification of positively selected sites (see Methods) indicated that a similar proportion of codons were called as positively selected in core (0.13%) and non-core/sub-core genes (0.12%).

**Fig 2:**
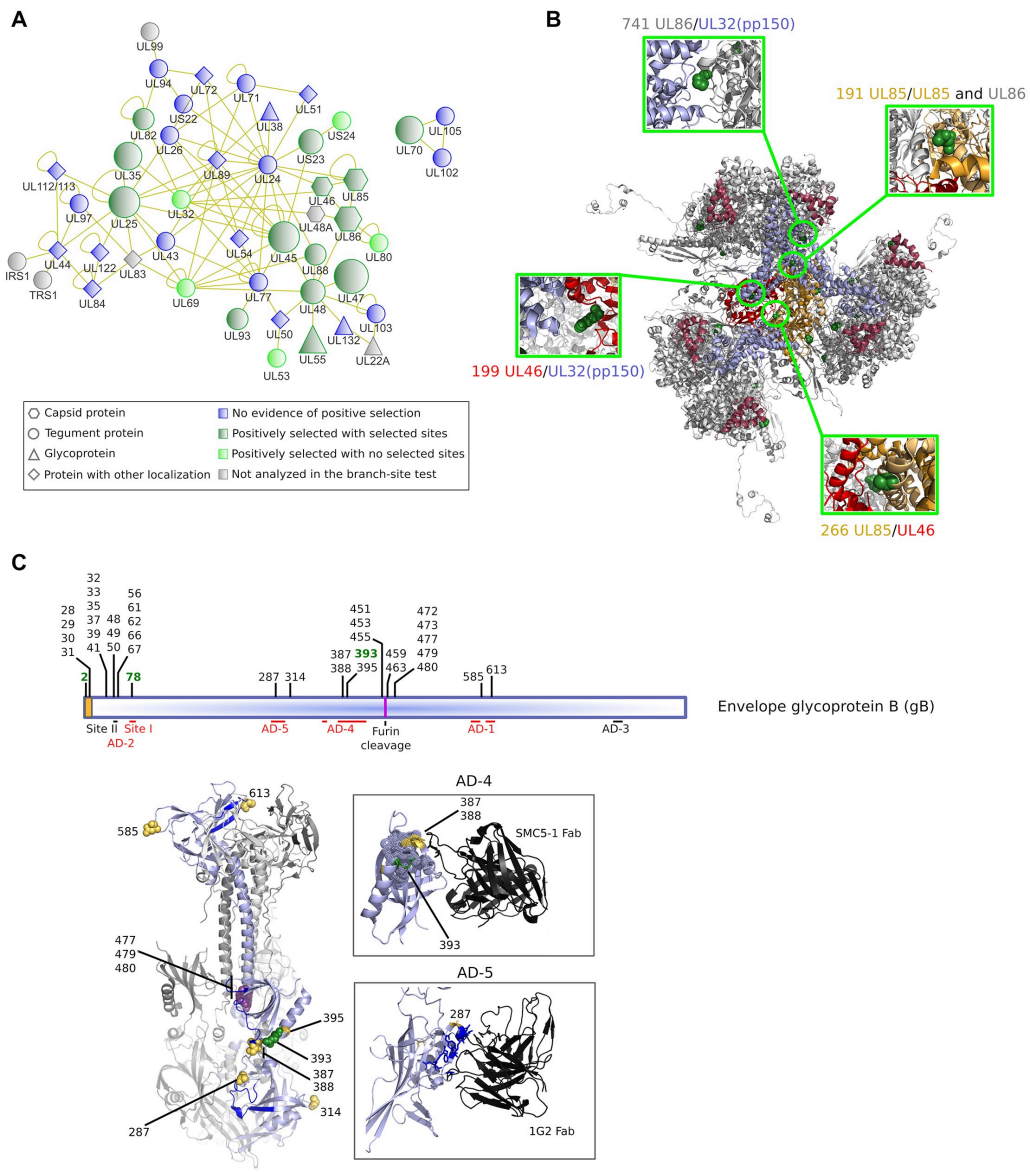
Episodic positive selection on the HCMV branch. **(A)** Protein-protein interaction network of HCMV proteins. Protein function/localization is coded with shapes, the selective patterns with colors. For positively selected proteins (forest green), node size is proportional to the number of detected positively selected sites. Protein-protein interaction data were derived from previous analyses [27,28] and from the VirHostNet database [29]. **(B)** Ribbon representation of the partial capsid structure of HCMV (PDB: 5VKU). Overview of the triplex structure (light orange: UL85 dimer, red:UL46), its surrounding major capsid proteins (UL86, grey), smallest capsid proteins (UL48A, maroon), and tegument proteins pp150 (UL32, light blue). Positively selected sites on the HCMV branch are shown as forest green spheres. Positively selected sites located at the interface with other molecules of the capsomer structure [30] are shown in the inserts. **(C)** Schematic representation of the gB protein. The signal peptide is in orange. Sites that were positively selected on the HCMV branch are shown in green, those identified by gammaMap are in black. The location of known antigenic determinants is reported [25, 26, 31–33]. Note that AD-2 is composed by two sites: site I (aa 64–84) contains epitopes recognized by neutralizing antibodies, site II (50–54) contains epitopes bound by non-neutralizing antibodies [25]. ADs that elicit neutralizing antibodies are marked in red. Several selected codons also flank the furin cleavage site. The structure of the gB trimer (PDB: 5CXF) is also shown [34]. For clarity, one monomer is represented in light blue, the other two are in gray. Regions encompassing neutralizing epitopes are shown in blue [26, 32, 33]. Selected sites are shown as spheres and color coded (green, selected on the HCMV branch; purple, selected in HCMV strains from at least two compartments; yellow, selected in HCMV strains from urine samples). In the two inserts, AD-4 and AD-5 in complex with neutralizing antibodies are shown. In AD-4 a hydrophobic pocket recognized by SMC5-1 (PDB: 4OT1) [26] is represented with dotted spheres. In AD-5, residues that directly contact 1G2 are marked in blue (PDB: 5C6T) [32].

Positive selection drove the evolution of three capsid proteins and of numerous tegument proteins that participate to an interaction network among themselves and with capsid components (Fig 2A). Four of the selected proteins (UL25, UL45, UL48, and UL69) represent major hubs in this network, which includes several components involved in virion assembly and maturation [22, 23].

Analysis of positively selected sites in *UL85*, *UL86*, and *UL46*, which encode structural constituents of the capsid, indicated that four of them are located at the contact interface with capsid components and/or tegument protein pp150 (UL32) (Fig 2B). In UL85, the two positively selected sites localize to the embracing arm, which has an important structural role in capsomer stability. In particular, residue 191 is located at the interface with UL86 and with the second UL85 molecule that compose the triplex structure; residue 266 is in close proximity to UL46 (Fig 2B). Residue 199 in UL46 and one selected site (residue 741) in UL86 are placed at the interface with the tegument protein pp150 (Fig 2B).

Only two glycoproteins, US11 and UL55 (glycoprotein B, gB) were among selection targets on the HCMV branch. gB mediates viral-host cell fusion and is a major target antigen [24]. Mapping of positively selected codons relative to known antigenic domains (AD) indicated that two of the three selected sites are located within ADs that elicit neutralizing antibodies [25, 26] (Fig 2C). Finally, positive selection was detected at genes that encode core viral enzymes such as UL70 (primase) and UL114 (uracil-DNA glycosylase) (S3 Table).

### Positively selected sites in *UL70* modulate viral replication

**Fig 3:**
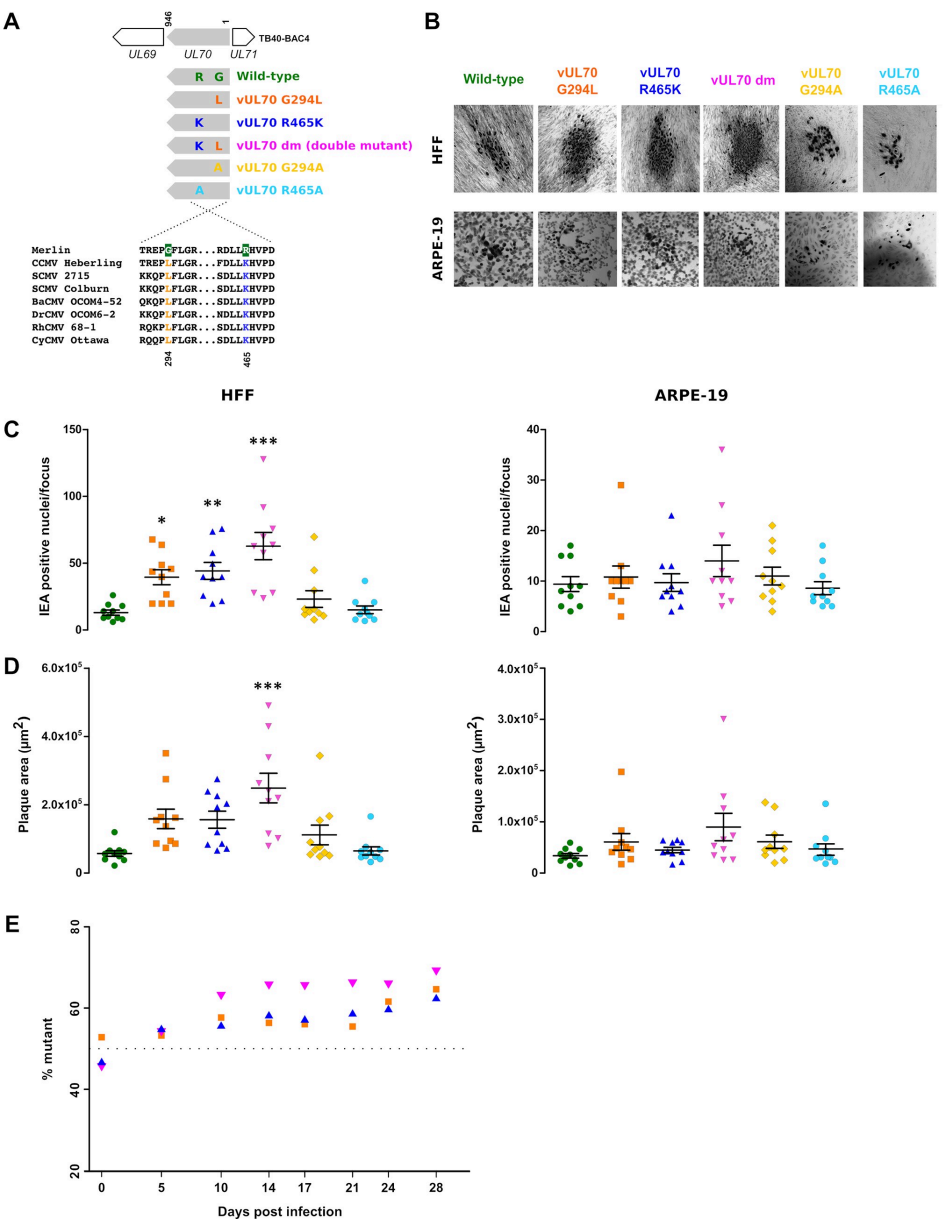
Phenotypic effect of positively selected sites. **(A)** Generation of vUL70 mutant strains. The HCMV *UL70* gene region is schematically represented together with the induced amino acid replacements at positively selected sites (G294 and R465). The alignment of a few catarrhini-infecting CMVs are shown. Wild-type and mutant viral strains are color-coded. (B**)** Representative light microscopy images of infectious foci are shown as seen in HFFs or ARPE-19 cells (5 days post infection). Original magnification: 20X. **(C)** IEA positive nuclei/focus. Each data point of the graph represents the number of IEA-positive nuclei of one focus at day 5 post infection. Bold horizontal lines depict the median number of IE antigen-positive cells/focus for each virus. One-way ANOVA followed by Tukey's post-hoc test was used for comparison of mutant viruses *versus* the wild-type (*P < 0.05; **P < 0.01; ***, P<0.001). **(D**) Plaque areas were calculated using ImageJ software. Horizontal lines represent mean values ± standard deviations. One-way ANOVA followed by Tukey's post-hoc test was used for comparison of mutant viruses *versus* the wild-type (***, P<0.001). **(E)** Head-to-head competition assays of three mutant viruses against the wild-type. HFFs were infected with a 1:1 ratio of wild-type and each mutant virus (MOI: 0.0005 PFU/ml). At different time points, total genomic DNA was extracted and subjected to deep sequencing. The relative proportion of mutant viral DNA is plotted. Each point represents the mean of two replicates.

Natural selection can only act on mutations that determine phenotypic variation. As a proof of concept that the sites we identified do modulate a viral phenotype, we decided to functionally characterize two selected variants in the viral primase (vUL70). In particular, we selected sites G294 and R465 because they are located in regions of high sequence conservation among CMVs (Fig 3A). Thus, point mutations that recapitulate the amino acid state observed in non-human infecting CMVs were introduced in the backbone of an infectious clone (TB40-BAC4) of the endotheliotropic HCMV strain TB40/E, either individually or in combination (Fig 3A and S4 Table). After “en passant” mutagenesis, viruses encoding wild-type vUL70 and mutant vUL70 (vUL70 G294L, vUL70 R465K, and vUL70 dm, for double mutant) were obtained (Fig 3A). As a control, mutant viruses carrying alanine at positions 294 and 465 were also generated (vUL70 G294A and vUL70 R465A) (Fig 3A).

To compare the cell-to-cell spreading efficiency of the mutant viruses to that of the wild-type parental strain, a focus expansion assay (FEA) was performed on HFFs (human foreskin fibrobalsts, where HCMV is typically propagated) and ARPE-19 cells (an *in vitro* epithelial cell model). Infections were performed with serial dilutions and cells were stained for HCMV IEA protein expression. The numbers of IEA positive foci in each cell dilution were comparable between the wild-type and the mutant viruses, in both HFFs and in ARPE-19 (S5 Table), suggesting that the overall replication dynamics of the viral strains was not affected by the UL70 mutations.

In agreement with previous works [35, 36], when HFFs were infected, all HCMV strains formed enlarged and diffused plaques throughout the culture (Fig 3B). In contrast, plaques appeared smaller and confined in ARPE-19 cells (Fig 3B). By measuring the plaque area and the number of IEA positive cells in each focus, we observed significant differences in the spreading capability in HFF among the mutant viruses. In fact, the vUL70 G294L, vUL70 R465K, and vUL70 dm mutants displayed a higher number of IEA positive HFFs in infected foci compared to the wild-type (Fig 3C and 3D). Conversely, none of the alanine-substitution mutants significantly affected FEA results compared to the wild-type virus. Consistently, the relative plaque area of the infected foci mirrored the IEA foci count, although a significant area increase was obtained for the double mutant only (Fig 3D). No significant difference among viruses was observed in ARPE-19 cells. The different phenotypes between the two cell types is most likely due to the fact that, compared to HFFs, ARPE-19 cells are less permissive to HCMV infection and support lower levels of virus release [37].

Given the results above, we assessed whether the three mutant viruses with amino acid substitutions observed in non-human infecting CMVs (vUL70 G294L, vUL70 R465K, and vUL70 dm) had higher fitness than the wild-type virus. To this aim, we performed head-to-head competition assays in HFFs, by infecting the cells with a 1:1 ratio of wild-type and each mutant virus with a multiplicity of infection (MOI) of 0.0005 PFU/ml. Viral growth was maintained for 28 days and the relative ratio of wild-type and mutant virus was assessed at different time points through deep sequencing. Results indicated that all mutants out-competed the wild-type, with their relatively proportions significantly increasing over time (all p values < 0.01, Cochran Armitage tests for trend). The effect was evident since the earlier time point (five days post infection), especially for the vUL70 R465K and vUL70 dm viruses (Fig 3E). At late time points, the relative proportions of all viruses tended to stabilize, most likely due to limiting conditions in cell culture. In general, the strongest competition over the wild-type was observed for the double mutant (Fig 3E). Calculation of the relative fitness gain (see methods) of the three mutant viruses yielded values of 2.5% (vUL70 G294L), 3.3% (vUL70 R465K), and 5.1% (vUL70 dm), in good agreement with the IEA foci count results.

Overall, these data indicate that the introduction of amino acid residues observed in non-human primate-infecting CMVs favors viral replication in human fibroblasts.

### Selective patterns in HCMV clinical isolates

Genome-wide evolutionary analyses of coding genes in HCMV isolates were previously reported [16, 38, 39]. These studies, however, applied methodologies that are best-suited to study inter-species diversity [16] (i.e., long-term evolutionary processes [40]), or used population genetics approaches to focus on within-host selection [39]. Alternatively, SNP enrichment was calculated to obtain an estimate of preferential selection in specific body compartments [41].

Herein, we applied a population genetics-phylogenetics approach to study the evolution of coding genes in HCMV strains from different compartments. Specifically, we used the gammaMap program [32], that jointly uses intra-species variation and inter-species diversity, to estimate the distribution of fitness effects (i.e. selection coefficients, γ, expressed as discrete categories from -500 to 100) along coding regions (see Materials and Methods and S6 Table). GammaMap is relatively insensitive to demography and recombination [42]. Analyses were performed for all coding genes in clinical isolates deriving from amniotic fluid, urine, and blood/plasma (S7 Table).

**Fig 4:**
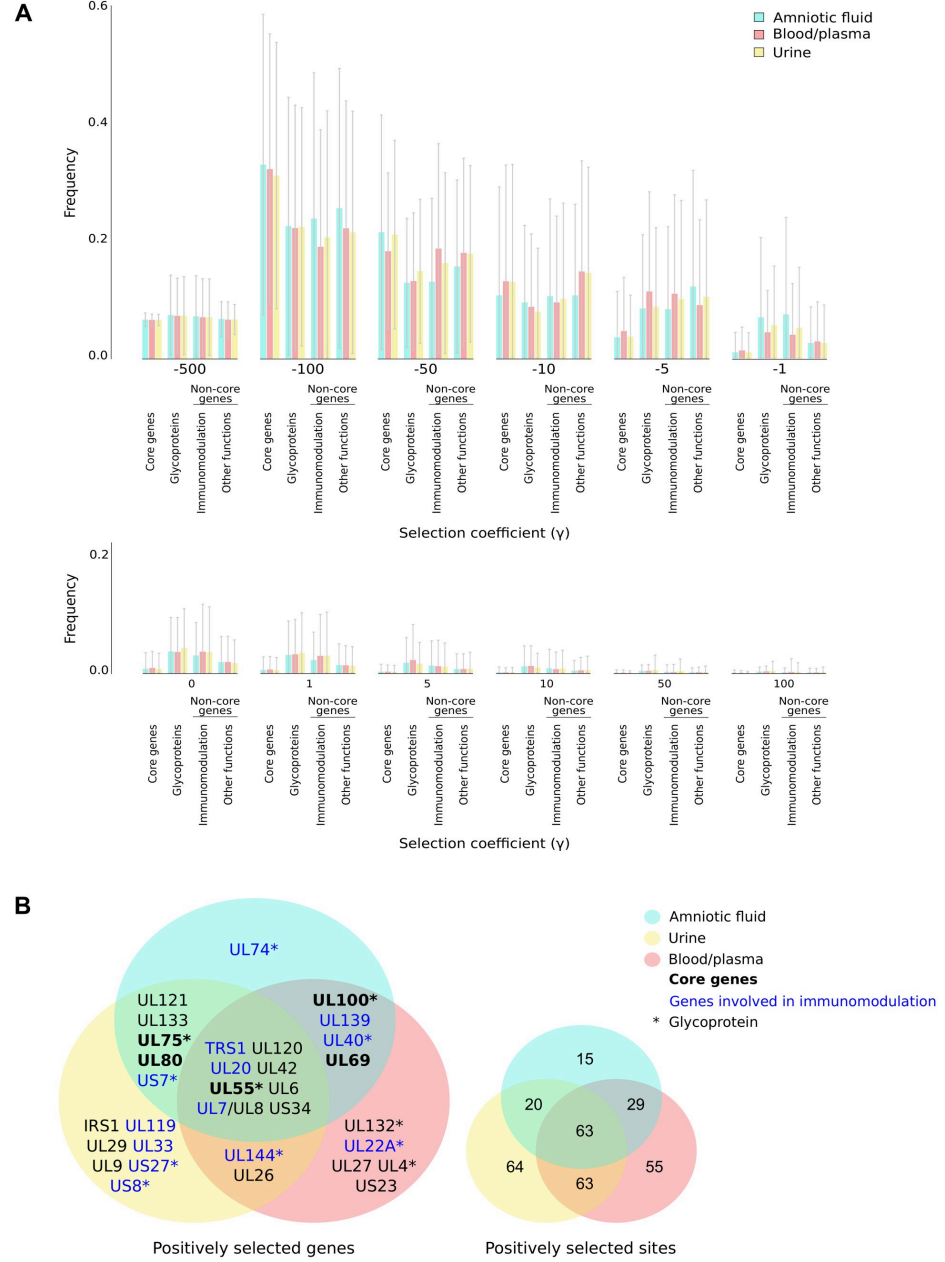
Selective patterns in HCMV clinical isolates from different compartments. **(A)** Distribution of selection coefficients (*γ)* for all coding genes of HCMV clinical isolates sampled from amniotic fluid, urine, and blood/plasma. Selection coefficients were calculated for all codons and genes were grouped on the basis of their conservation (core, non-core/sub-core) and of the functional classification of the encoded protein (glycoprotein, both core and non-core; immunomodulation, non-core only). Large error bars are caused by the difficulty in determining the relative frequency of similar selection coefficients. **(B)** Venn diagrams of positively selected genes and sites in HCMV isolates.

Overall, we observed a strong preponderance of codons evolving under negative selection (−500 ≤ *γ* ≤ −1) and a non-negligible fraction of positive selection (*γ* > 1) signals. No substantial differences in the distribution of selection coefficients was observed across the three body compartments (Fig 4A). The selective patterns were also similar between genes that were or were not called as positively selected in the branch-site test, suggesting that the evolutionary processes that drive host adaptation do not necessarily parallel those that occur at the intra-species level (S3 Fig). Core genes, however, showed a larger proportion of strongly constrained codons compared to non-core/sub-core genes, whereas the immunomodulation and glycoprotein gene functions showed comparatively fewer sites under negative selection and more with positive selection signals (Fig 4A). Despite these differences, most genes had a proportion of codons evolving under strong to moderate negative selection (*γ* < -10) (S4 Fig).

To define the signals of positive selection, we estimated codon-wise posterior probabilities for each selection coefficient. We called a codon as positively selected if its cumulative posterior probability of *γ* ≥ 1 was > 0.80. A total of 32 genes (5 core, 27 non-core) were found to be positively selected (i.e., to have at least one selected codon) in at least one compartment (Fig 4B, S8 Table). Although there was a large overlap among compartments, isolates from urine and blood/plasma had more abundant and more divergent signals of positive selection compared to those from amniotic fluid, in which the only compartment-specific signal was at *UL74* (glycoprotein O, gO) (Fig 4B).

Among positively selected genes, glycoproteins were significantly enriched (Fisher exact test with FDR correction, p = 0.0195). A tendency for enrichment was also observed for genes with immunomodulatory function (Fisher exact test with FDR correction, p = 0.0514), but not for genes involved in cell tropism (Fisher exact test with FDR correction, p = 1).

We detected positively selected sites in nine genes (*UL33*, *UL4*, *UL55*, *UL74*, *UL75*, *UL100*, *UL119*, *UL132*, and *US27*) coding for viral envelope proteins. The majority of such sites (88.3%) are located in protein regions exposed on the virion surface (S5 Fig and S8 Table). Although exposed regions account for a large portion of the protein sequences, the number of selected sites was significantly higher than expected (Binomial test, p value = 0.00925). Likewise, eight genes (*UL6*, *UL7/8*, *UL9*, *UL42*, *UL120*, *UL121*, *UL139*, and *UL144*) that encode proteins expressed at the host cell membrane were targeted by positive selection and most sites (90.8%) were in the extracellular domains (Binomial test, p value = 0.001955) (S5 Fig and S8 Table).

**Fig 5:**
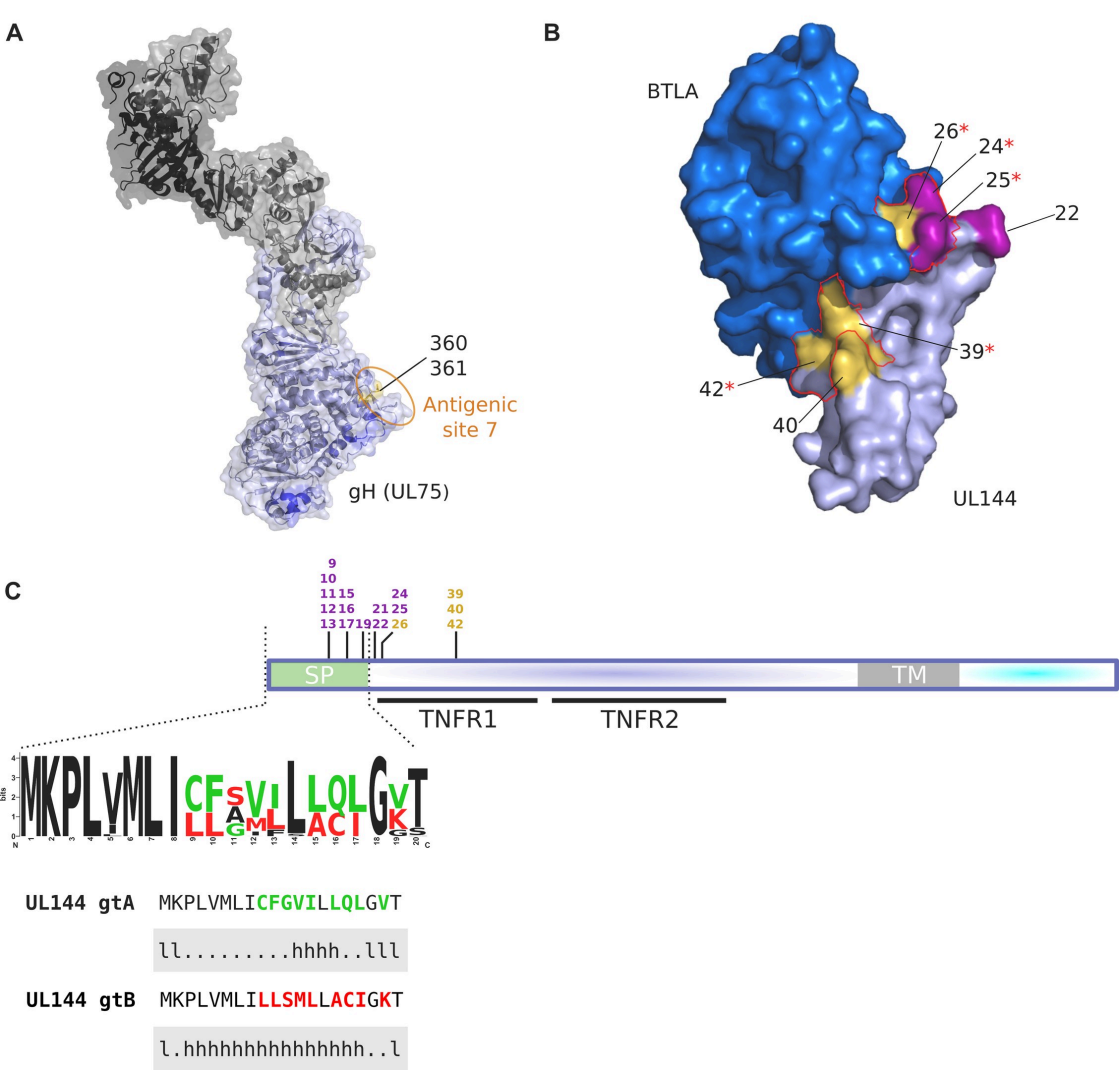
Positively selected sites in clinical isolates. **(A)** Structure of the HCMV pentamer (PDB: 5VOC) [45]. gH is shown in light blue, gL, UL128, UL130, and UL131 in shades of grey. The location of known antigenic sites is shown in blue [46–48]. Positively selected sites in urine-derived samples are shown in yellow and are surface-exposed. These sites localize to antigenic site 7 [43]. **(B)** 3D structure of HCMV UL144 (light purple) in complex with BTLA (blue) (PDB: 6NYP). Positively selected sites in urine-derived samples and in at least two different compartments are shown in yellow and purple, respectively. Residues critical for BTLA binding are highlighted with a red outline. Red asterisks denote positively selected sites directly involved in BTLA binding [49]. **(C)** Schematic representation of the UL144 protein. Positively selected sites are color coded as in (B). The location of tumor necrosis factor receptor/nerve growth factor receptor repeats (TNFR1 and TNFR2) is reported. The signal peptide (SP) is in green. The sequence logos recapitulate the amino acidic variation observed in SPs of HCMV clinical samples. Information content (bits) is plotted as a function of amino acid position. Sequence logos were generated with WebLogo [50]. The amino acid state of positively selected sites that define the two SPs are reported (gtA, green; gtB, red). The secondary structure prediction (l, loop; h, helix), generated with PredictProtein [51], is also shown below both sequences.

Overall, these findings suggest that a major selective pressure on these genes is exerted by the host immune system. For instance, several selected sites in gB map within ADs (Fig 2C), and in gH (UL75) positively selected sites localize to a structural epitope within antigenic site 7 [43] (Fig 5A). Additional details on the location of positively selected sites, sequence polymorphism, and ADs for gB, gH, gM and gO are reported in S6 Fig. As for UL144, positively selected sites are directly involved in BTLA (B and T lymphocyte attenuator) binding (Fig 5B) [44].

In UL144, nine sites were also located in the short signal peptide (SP) (Fig 5C). More generally, we found evidence of positive selection in the SPs of several proteins (UL9, UL20, gB, gH, UL132, US7, in addition to UL144) (S5 Fig and S8 Table). Overall, 9% of the positively selected sites we detected were located in SPs, a proportion significantly higher than expected (Binomial test, p value = 0.001154).

### Positively selected sites in the UL144 signal peptide modulate intracellular trafficking and the timing of cleavage by the signal peptidase

Given the results above, we decided to functionally characterize the effects of selected variants within the UL144 SP. We focused on UL144 because its SP was strongly targeted by selection and because UL144 is a membrane glycoprotein with important immunomodulatory functions [52, 53]. The nine sites in the SP were called as positively selected in blood/plasma and urine samples; their amino acid states differ among previously described UL144 genotypes [54].

We analyzed the intracellular trafficking of two UL144 sequences: one that is identical to the protein encoded by the Merlin strain and corresponds to genotype A (gtA) and one that carries amino acid replacements at the 9 positively selected sites and corresponds to the SP of genotype B (gtB). These two variants have similar representation among clinical isolates (Fig 5C). The prediction of protein secondary structure indicated a shorter helix domain for the gtA SP compared to gtB (Fig 5C). This feature was reported to facilitate the cleavage by the signal peptidase in *E*. *coli* [55] and changes in the helix structure of the HIV-1 envelope glycoprotein (Env) SP modulate the timing of its cleavage [56].

**Fig 6:**
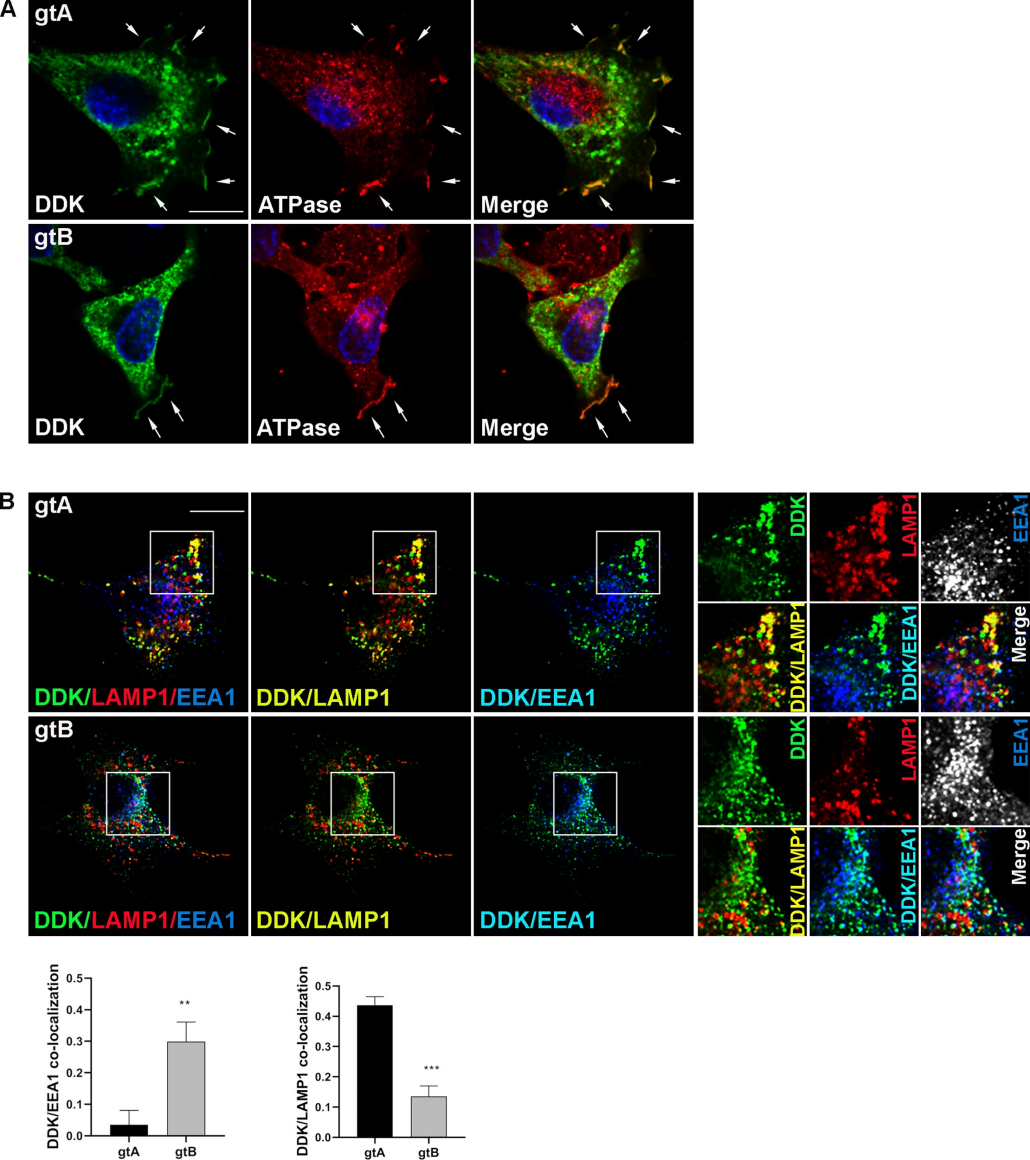
Functional analysis of positively selected sites in the UL144 SP. HeLa cells were transfected with pCMV6-UL144 gtA and pCMV6-UL144 gtB. Twenty-four hours later, cells were fixed and immunostained. **(A)** Localization at the plasma membrane. Cells were stained with antibodies against the DDK tag (green) and the plasma membrane protein sodium potassium ATPase (red). Nuclei were counterstained with DAPI. Arrows indicates localization at the plasma membrane. Scale bar: 10 μm. **(B)** Localization in the endo-lysosomal compartment. Cells were immunostained with antibodies against the DDK tag (green), the lysosomal marker LAMP1 (red) and the early endosomal marker EEA1 (blue). Co-localization of DDK with LAMP1 (yellow) or EEA1 (light blue) is shown in the merge images. The small panels show an higher magnification of the area indicated in the squares. Scale bar: 10 μm. Pearson’s correlation coefficients for DDK/LAMP1 and DDK/EEA1 co-localization are reported in the graphs as mean ± SEM (*t* test; n > 30) (**, P < 0.01; ***, P < 0.001).

The two UL144 variants with a DDK tag (hereafter referred to as gtA and gtB) were transiently expressed in HeLa cells and detected with an anti-DDK antibody (green). Twenty-four hours after transfection, both gtA and gtB reached the cell membrane as expected (Fig 6A), but were also differentially detected in intracellular vesicles (Fig 6B). Indeed, while gtB mainly co-localized with the early endosomal marker EEA1 (blue, co-localization light-blue), the gtA variant showed predominant co-localization with the lysosomal marker LAMP1 (red, co-localization yellow) (Fig 6B). Endosomes represent the first compartment of the endocytic pathway for the sorting of internalized protein from the plasma membrane, that terminates in the lysosomes. The differences in the localization of the two UL144 variants in these compartments (Fig 6B) therefore suggest a sorting delay of gtB compared to gtA.

**Fig 7:**
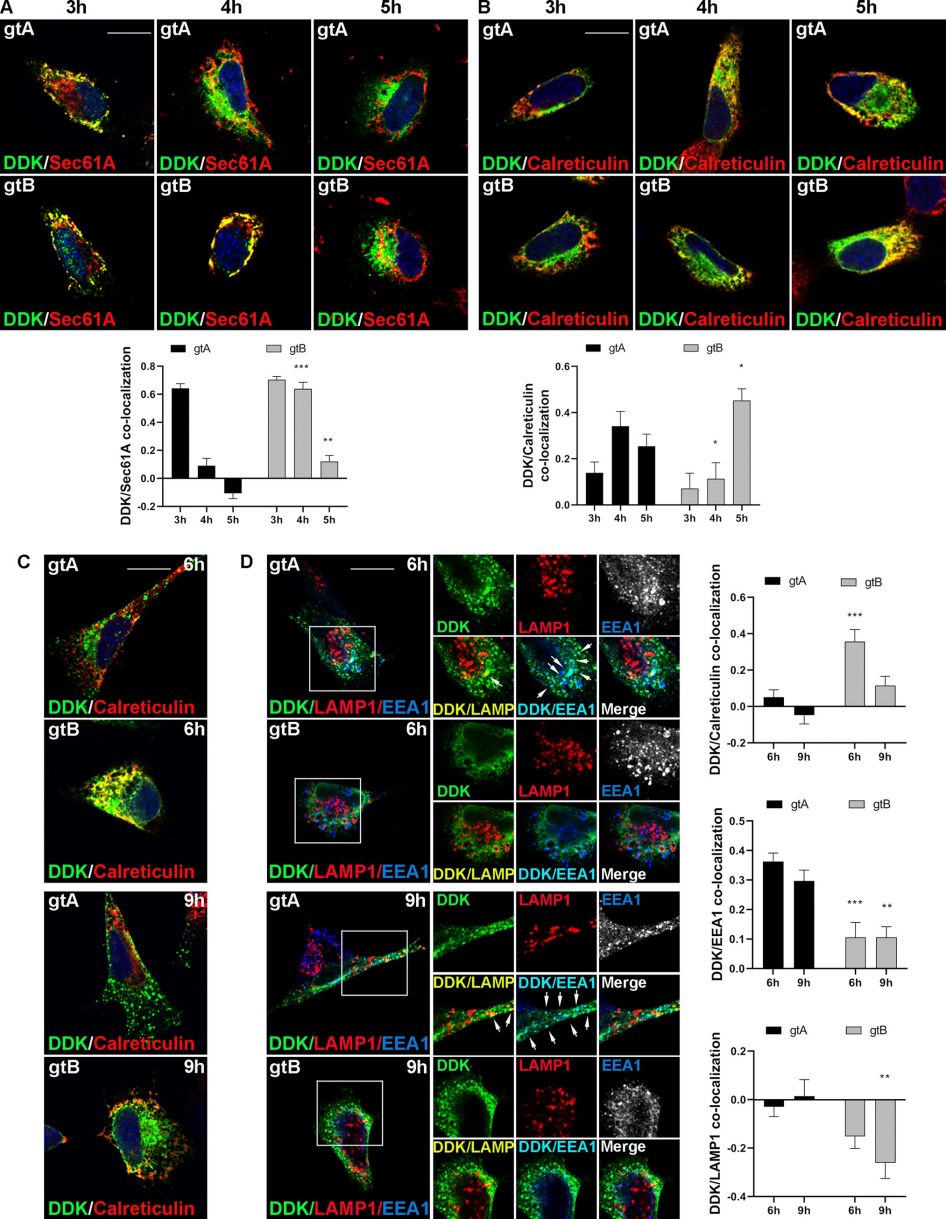
Time-course analysis of UL144 gtA and gtB intracellular trafficking. HeLa cells were transfected with pCMV6-UL144 gtA and pCMV6-UL144 gtB and fixed at different time points. Three, four and five hours post transfection cells were immunostained with anti-DDK (green) and anti-Sec61A (red) Abs (**A**) or with anti-DDK (green) and anti-Calreticulin (red) Abs (**B**). Nuclei were counterstained with DAPI. Yellow indicates co-localization. Scale bar: 10 μm. Pearson’s correlation coefficients for DDK/Sec61A and DDK/Calreticulin co-localization are reported in the graphs as mean ± SEM (two way ANOVA; n > 25) (*, P < 0.05; **, P < 0.01; ***, P < 0.001). (**C**) Six and nine hours post transfection cells were immunostained with anti-DDK (green) and anti-Calreticulin (red) antibodies. Nuclei were counterstained with DAPI. Yellow indicates co-localization. Scale bar: 10 μm. (**D**) Six and nine hours post transfection cells were immunostained with anti-DDK (green), anti-LAMP1 (red) and anti-EEA1 (blue) antibodies. The small panels show an higher magnification of the area indicated in the squares. The arrows indicate co-localizing vesicles. Scale bar: 10 μm. Pearson’s correlation coefficients for DDK/Calreticulin, DDK/EEA1 and DDK/LAMP1 co-localization are reported in the graphs as mean ± SEM (two way ANOVA; n > 25) (**, P < 0.01; ***, P < 0.001).

To monitor the differences in the cellular trafficking of the gtA and gtB variants, we analyzed their localization at different times after transfection. Membrane proteins are commonly targeted to the endoplasmic reticulum (ER) by the signal peptide through the Sec61 translocon [57]. We therefore evaluated the co-localization of gtA and gtB with the translocon complex (Sec61a) and with calreticulin, an ER luminal resident protein, at 3, 4 and 5 hours after transfection. Three hours after transfection, the two variants showed a similar co-localization with Sec61a (red) (Fig 7A), indicating that the initial contact with the translocon occurs with the same efficiency. Conversely at 4 hours, while the gtA variant moved to other ER compartments mainly co-localizing with calreticulin (red), gtB was still associated with Sec61a and its co-localization with calreticulin was delayed compared to gtA (Fig 7A and 7B), indicating that gtB is retained for a longer time by the translocon complex. Differences in the sorting dynamics of the two variants were also evident at 6 and 9 hours post transfection, when we analyzed the co-localization of the UL144 variants with the early endosomal marker EEA1 (blue) and with the lysosomal marker LAMP1 (red). At 6 hours post transfection, gtA was mainly found in early endosomes, whereas most of gtB was still located within the ER and only a minor co-localization with EEA1 was detected (Fig 7C and 7D). None of the two variants localized to the lysosomes at this time point (Fig 7D). The delayed processing of gtB remains unchanged at 9 hours post transfection (Fig 7C and 7D) and eventually explains the differential localization observed at 24 hours.

These results, also confirmed in HEK-293 cells (S7 Fig), indicate that the selected sites in the UL144 SP are sufficient to alter the intracellular trafficking of the glycoprotein.

**Fig 8:**
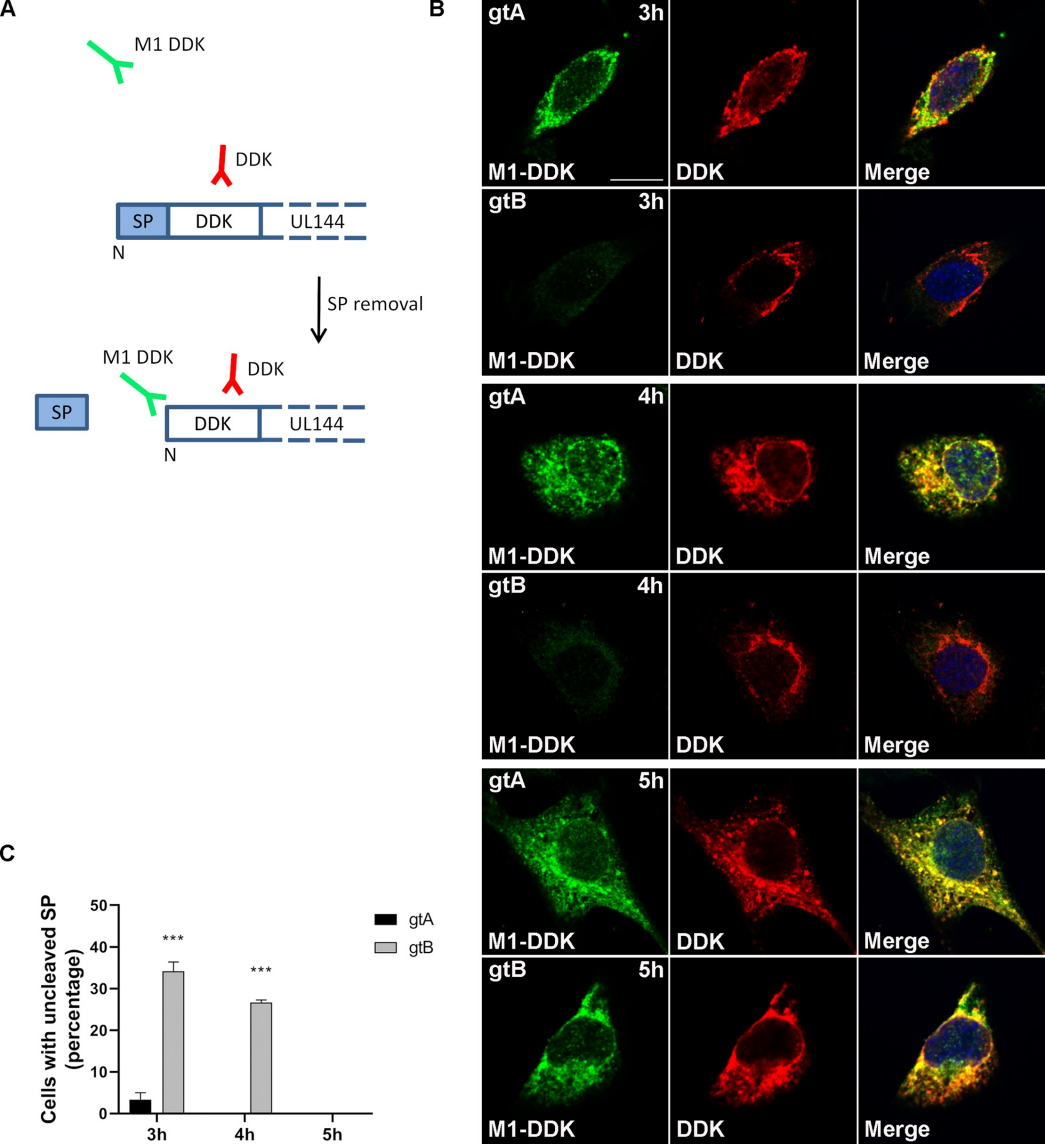
Time-course analysis of signal peptide cleavage. (**A**) Schematic representation of the vectors used in the experiment. To analyze the removal of the signal peptide from the gtA and gtB variants, a DDK tag was cloned immediately downstream the SP. The DDK tag was detected by using two antibodies: an anti-M1-DDK Ab (green signal) that recognizes only the free N-terminal end of the DDK tag (generated by the cleavage of the SP), and an anti-DDK Ab (red signal) that recognizes the tag independently of its position in the protein and stains all the cells transfected with UL144 variants. (**B**) HeLa cells were transfected and fixed at different time points. Three, four, and five hours post transfection cells were immunostained with anti-M1-DDK and anti-DDK Abs. Nuclei were counterstained with DAPI. Yellow in the merge images indicates co-localization. Scale bar: 10 μm. (**C**) Cells only stained with the anti-DDK Ab (red only, indicating an uncleaved SP) were counted, normalized to the total number of analyzed cells and reported as percentage (two way ANOVA; n >60) (***, P < 0.001).

To determine whether the delayed trafficking of the UL144 gtB variant is due to late cleavage of the SP, we generated two expression vectors carrying the DDK tag immediately downstream the gtA and gtB SPs (Fig 8A). Immunofluorescence analysis was then performed 3, 4 and 5 hours after transfection using two antibodies: the anti-M1-DDK antibody, that specifically recognizes the free N-terminal end of the DDK epitope and thus stains the protein only after SP cleavage [58], and the anti-DDK antibody used above, which binds the epitope irrespective of its sequence context (Fig 8A). At 3 hours after transfection, while almost all the cells transfected with gtA were positive for both M1-DDK (green) and DDK (red) staining, indicating SP removal, the 35% of the cells transfected with gtB still presented the SP and were therefore stained by the anti-DDK antibody only (Fig 8B and 8C). This delay in the removal of the SP from gtB was also maintained at 4 hours after transfection. It was not until 5 hours post transfection that the SP was cleaved from the gtB variant in all the analyzed cells.

## Discussion

CMVs display a remarkable host-specificity even *in vitro—*i.e., in the absence of the host adaptive immune response. Such specificity is mainly driven by post-entry events [59–63]. In the case of HCMV, chimpanzee primary fibroblasts allow low-level viral replication [64], but other non-human primate cells do not support the production of infectious virions [59–61]. This clearly implies that HCMV must have adapted to efficiently complete its infectious cycle in human cells. Our data indicate that, although core genes were generally more constrained during primate CMV evolution, a number of them were targeted by positive selection during HCMV speciation. We found evidence of selection at capsid components and at numerous tegument proteins that drive important steps in virion maturation and assembly. Such steps are orchestrated by an interplay of interactions among viral- and host-encoded proteins [23], suggesting that the selection signals we identified represent polygenic adaptation of HCMV to replication in human cells.

Evidence of selection was also detected at two core viral enzymes, including the primase, which we decided to characterize experimentally. We found that UL70 mutations that recapitulate the amino acid state observed in non-human infecting CMVs display increase cell-to-cell spreading efficiency in human fibroblasts. Competition experiments also indicated that mutant viruses have higher fitness than a virus carrying the same amino acid states as circulating HCMV strains. Although this finding may seem counter-intuitive, it is known that HCMV has evolved strategies to reduce or control its replication rate and, most likely, its virulence. Such strategies were referred to as “temperance” by Dunn and coworkers, who performed a large-scale analysis of the effect of HCMV gene mutations on viral growth [18]. They found that deletion of some viral genes (e.g., *UL10*, *UL23*, and *US16*) increased rather than decreased replication in specific cell types and suggested that this temperance effect is necessary to promote long-term viral co-existence with its host [18]. In analogy, we found that the cell-to-cell spreading effect of UL70 mutations is cell-type specific, as only minor, non significant differences were observed in an epithelial cell type. Nonetheless, additional experiments will be required to assess whether adaptive mutations in the *UL70* gene arose during HCMV evolution as temperance factors.

Analysis of the distribution of fitness effects in HCMV clinical isolates clearly indicated that the selective events that occurred during HCMV speciation (i.e., most likely as a result of human adaptation) differ considerably from the selective patterns that govern intra-species variation (i.e., ongoing or recent adaptive events). The selective pressure exerted by the host immune system has likely played a major role in the shaping of genetic diversity among circulating strains. In line with previous findings, we found that glycoproteins and proteins involved in immunomodulation tend to be less constrained and to display the strongest signals of positive selection [16, 38, 41]. This effect was observed in all compartments, that also showed a similar overall distribution of selection coefficients. This is in contrast with previous suggestions that isolates from vascular compartments are more selectively constrained than those from urine [41]. Samples from blood/plasma and urine had more abundant and more diverse selection signals compared to those isolated from amniotic fluid, a finding that is not necessarily due to the specific compartments, but may depend on the number of available isolates and/or on their diversity. In fact, several positively selected genes and sites were shared among compartments, revealing limited tissue-specific adaptive events.

Some of the positively selected genes we identified using the population genetics-phylogenetic approach were previously found to represent positive selection targets during intra-host evolution (*UL7*, *US7*, *UL74*, *US27*, *UL20*, *UL132*, *UL80*, *UL55*) [39] or to display an excess of nonsynonymous polymorphism in plasma samples (*UL55*, *UL74*, *UL75*) [41]. In gB (UL55) and gH (UL75), most positively selected sites were located within ADs and, in the case of UL144, several sites were within the interaction surface with BTLA, again suggesting immune system-driven selective pressure. gB and gH are highly immunogenic and represent major targets of neutralizing antibodies. Extensive analysis of antibody responses indicated that different ADs elicit neutralizing antibodies in a variable proportion of naturally infected or immunized individuals [65–67]. In some instances, such as the AP86 epitope in the N-terminus of gH, where no positively selected sites were detected, variability among HCMV strains is known to modulate the antibody response [68]. In the case of gB ADs, it is presently unclear whether viral diversity affects neutralization capacity. Recently, a correlation was observed between anti-AD-2 serum titers and protection from viremia in a vaccination trial [69], although only a proportion of infected individuals developed AD-2 neutralizing responses. The majority of positively selected sites we found in AD-2 map to a site which is known to elicit non-neutralizing antibodies, raising questions about their adaptive significance in terms of immune evasion. A possibility is that, as previously shown for AD-1, a competition between neutralizing and non-neutralizing antibodies at site I and site II represent a mechanism to evade humoral responses [70]. Thus, although we found a very good correspondence between the location of ADs and selected sites, the relevance of the latter for immunoevasion and, ultimately, vaccine design, remains to be evaluated.

In gB, we also detected several selected sites across the furin cleavage site. Using a different approach, variants in this region were previously shown to be targeted by positive selection and to modulate the kinetic of furin cleavage [71]. As for other herpesviruses with furin cleavable gBs, proteolytic processing by furin is dispensable for HCMV growth in cell culture [72–75]. However, because the furin cleavage site is evolutionary conserved in several members of the *Herpesviridae* family, processing may confer some advantages under specific circumstances or in specific cell types. For instance, in other herpesviruses, loss of gB cleavage decreases viral cell-to-cell spread, sometimes in a cell-type dependent manner [73, 74, 76, 77]. Cell-to-cell spread is a typical feature of HCMV clinical isolates [78], suggesting that variation at the selected sites might modulate furin cleavage and viral dissemination in specific cell types or tissues.

An unexpected finding was the identification of SPs as major targets of positive selection in HCMV isolates. Although they were considered for a long time as simple and interchangeable targeting signals, emerging evidence indicate that SPs can regulate important steps in protein processing, including translocon interaction efficiency, glycosylation patterns, and folding [79]. However, few naturally occurring variants in the SPs of viral proteins were characterized. Herein we show that positively selected sites within the UL144 SP modulate the dynamics of SP processing and of protein sorting. In fact, we show that the delayed trafficking of UL144 gtB compared to gtA is due to late cleavage by of the SP. This is in line with experiment on engineered and mammalian proteins showing that the SP sequence can determine the timing of its cleavage [80]. Following translocation through the Sec61 channel, the SP is inserted into the ER membrane and, for most proteins, co-translationally cleaved [81]. However, some SPs are cleaved post-translationally. This is the case of another HCMV protein, US11, an ER-resident protein: the sequence of the US11 SP determines its late removal, although the functional significance of this unusual processing is still unknown [82]. The best-studied case of late SP removal, however, is that of the HIV-1 Env protein. The Env SP acts as a membrane tether for at least 15 min after protein synthesis and its cleavage occurs only after initial folding of the ectodomain [83]. The SP sequence is sufficient to dictate late cleavage [56], which, in turn, determines correct protein folding and eventually modulates Env antigenic properties and glycosilation patterns, as well as Env incorporation into virions and HIV-1 infectivity [56, 84, 85]. Because the Env SP is highly variable, it was suggested that, although not present in the mature protein, it is subject to antibody-mediated immune pressure [84]. Notably, we also detected positively selected sites in the SPs of gB and gH, which represent major targets of neutralizing antibodies and promising candidates for vaccine development. An alternative possibility is that the changes in sorting dynamics and SP cleavage timing affect other protein functions, such as the efficiency of UL144 binding to its cellular partner or the amount of mature protein that is delivered to functionally relevant location. Other experiments will be needed to disentangle these possibilities.

Molecular evolution analyses can provide information on the location and nature of adaptive changes in genomic regions, thus highlighting the presence of functional variation either at the inter- or intra-species level. We performed a two-tier analysis of HCMV evolution, by describing selective events that occurred during HCMV speciation and by identifying more recently emerged adaptive variants in clinical isolates. We provide proof-of-concept validation of the functional effect of positively selected sites in two viral genes. Thus, we used evolutionary information to generate experimentally-testable hypotheses concerning the functional effect of HCMV diversity, eventually generating a catalog of candidate modulators of viral phenotypes.

## Materials and methods

### Sequences and alignments

Viral genome sequences were retrieved from the National Center for Biotechnology Information database (NCBI, http://www.ncbi.nlm.nih.gov/). Only complete genome sequences were included in this study (S1 and S7 Tables). For each genome, we retrieved coding sequences corresponding to the 169 ORFs annotated for the Merlin strain (NC_006273). Orthology was inferred according to previously reported analyses and genome annotations, as well as by applying a phylogenetic approach to the largest gene families (see below) [14, 86–89]. Gene alignments were generated using MAFFT (version 7.392) [90], setting sequence type as codons and using default parameters; unreliably aligned codons were filtered using GUIDANCE2 [91] with a score of 0.90 [92]. The resulting alignments were manually inspected.

Whole genome alignments were obtained using Progressive MAUVE 2.3.1 [93, 94], a program designed to construct multiple genome alignments in the presence of large-scale changes such as deletions, rearrangements and inversions. MAUVE identifies and aligns regions of local collinearity (locally collinear blocks, LCBs). Each LCB is a region of sequence homology shared by two or more of the genomes being aligned. MAUVE was run using default parameters.

### Coding gene annotations and protein interaction network

Classification based on gene conservation was obtained from previous works [7, 10]. Functional classification was obtained from a survey of HCMV proteins [11]. For protein localization, information was obtained from gene ontology classifications (component category) via the QuickGO server (https://www.ebi.ac.uk/QuickGO/). Because the same gene can be associated with different functional classification and/or GO component terms (i.e., terms are not independent), we compared genes associated with a given term with those that are not associated to it. Terms were included only if they had more than five contributing genes.

Growth phenotypes were obtained from Van Damme et al. [11]. Phenotypes were made independent by considering as essential (E) only genes that were unequivocally described as such—e.g., genes annotated as essential/growth defective (GD) were assigned to the GD category- and by ascribing to the GD category any gene for which growth defect have been described at least once. Finally, the enhanced growth phenotype (2 genes) was collated to the dispensable (D) category.

Data on protein-protein interactions were derived from two yeast two-hybrid analyses that tested binary interaction between more than 30 proteins each [27, 28]. Additional protein-protein interaction data were derived from the VirHostNet database (http://virhostnet.prabi.fr) [29]. All interactions (physical associations) between HCMV-encoded proteins were retrieved from VirHostNet, irrespective of the viral strain to which they referred. Data from the two studies and from VirHostNet were merged and a network was visualized using Cytoscape version 3.6.0 [95].

### Orthology assessment for large gene families

In order to make a reliable orthology analysis of the four largest gene families (*RL11*, *US22*, *US6*, and *US12* families), we used HCMV proteins belonging to these families to query the genomes of primate CMVs using different bioinformatic tools. Specifically we used: 1) protein BLAST (https://blast.ncbi.nlm.nih.gov/Blast.cgi); 2) the Conserved Domain Search service (CD-Search), a web-based tool for the detection of structural and functional domains in protein sequences from the NCBI's Conserved Domain Database (CDD) server [96]; 3) the HMM-HMM–based lightning-fast iterative sequence search (HHblits), which uses a profile-profile alignment prefilter enabling fast, iterative and accurate sequence searches [97]. All identified homologs were aligned using MAFFT (version 7.392) using default parameters. Phylogenetic trees were constructed using RAxML (version 8.2.12, PROTGAMMAJTT matrix) with 1000 bootstrap replicates [98]. Orthologous genes were inferred on the basis of the tree topology (sequences clustering by viral species) and of bootstrap values (support higher than 90).

### Selective patterns in catarrhini-infecting CMVs

To obtain an overview of the selective patterns of primate CMV coding genes, we analyzed a viral phylogeny composed of 11 strains including 3 CMVs that infect great apes and 8 that infect either African or Asian Old World monkeys (S1 Table). Only one-to-one orthologs were included in the analysis. Alignments that, after GUIDANCE filtering, had less than 250 aligned nucleotides were discarded (S2 Table). The average dN/dS parameter was calculated using the single-likelihood ancestor counting (SLAC) method [99]. Inputs were the multiple sequence alignments and trees generated with the phyML program (version 3.1). For the latter, we applied a General Time Reversible (GTR) model plus gamma-distributed rates and 4 substitution rate categories, a fixed proportion of invariable sites, and a BioNJ starting tree [100].

Differences in dN/dS among catarrhini-infecting CMVs genes grouped on the basis of gene conservation and gene dispensability were evaluated using Kruskal-Wallis tests. Nemenyi tests with χ2 distribution to account for ties were used as post-hocs. Differences in dN/dS among genes grouped on the basis of functional classification and protein localization were evaluated by Wilcoxon Rank-Sum tests. FDR correction was applied to account for multiple tests. These calculations were performed in the R environment using the PMCMR package [101].

### Detection of positive selection in the HCMV lineage

Analyses were performed on a phylogeny that included all fully-sequenced catarrhini-infecting CMVs (n = 16) and 25 HCMV clinical isolates with no history of passaging in cell culture. Strains were selected to derive from different tissues and from different countries (S1 Table).

Phylogenetic trees were reconstructed using phyML (version 3.1). Each alignment was screened for the presence of recombination using GARD [102], a genetic algorithm implemented in the HYPHY suite (version 2.2.4). This method uses phylogenetic incongruence among segments in the alignment to detect the best-fit number and location of recombination breakpoints. The statistical significance of putative breakpoints is determined through Kishino-Hasegawa (HK) tests. When evidence of recombination was detected (p-value<0.01), the coding alignment was split on the basis of the recombination breakpoints and sub-regions were used as the input for molecular evolution analyses. Recombination breakpoints were identified in 12 genes: *UL29*, *UL44*, *UL48*, *UL54*, *UL55*, *UL56*, *UL57*, *UL74*, *UL75*, *UL123*, *US23 and US28*. Only resulting alignments that, after GUIDANCE filtering had a length ≥ 250 nt were considered for subsequent analyses.

To search for episodic positive selection on the HCMV branch, we applied the branch-site likelihood ratio tests from codeml (“test 2”) [21]. In this test, branches are divided *a priori* into foreground (those to be analyzed for positive selection, in this case the branch leading to the 25 HCMV isolates) and background lineages (in this case all other branches), and a likelihood ratio test is applied to compare a model (MA) that allows positive selection on the foreground lineages with a model (MA1) that does not allow such positive selection. Twice the difference of likelihood for the two models (ΔlnL) is then compared to a χ2 distribution with one degree of freedom [21]. The analyses were performed using an F3X4 codon frequency models. An FDR correction was applied to account for multiple tests.

To identify sites evolving under positive selection on HCMV lineage we used the BEB analysis from MA (with a cutoff of 0.90) and the Mixed Effects Model of Evolution (MEME) (with the default cutoff of 0.1) [103]. MEME allows the distribution of nonsynonymous substitution/synonymous substitution rate (dN/dS, also referred to as ω) to vary from site to site and from branch to branch at a site. To limit false positives, only sites confirmed by both methods were considered as positively selected.

### Analysis of selective patterns in HCMV isolates

HCMV genomes were derived from the NCBI database (NCBI, http://www.ncbi.nlm.nih.gov). Based on the number of available sequences, we analyzed HCMV isolates derived from amniotic fluid, urine and blood/plasma. Most of these latter derive from transplant recipients, whereas little information on the clinical history of the infected subjects was available for the other isolates (S7 Table). Only isolates that were directly sequenced with no *in vitro* passages were included. When stop codon/frameshift mutations were identified (e.g., in *UL9*), the gene of the isolate was removed from analysis.

For all genes, a HCMV outgroup sequence was reconstructed by maximum-likelihood from a balanced subset of no/low passaged HCMV strains derived from different compartments (S6 Table). Maximum-likelihood inference was obtained with FastML [104]. The distribution of fitness effects (DFE) along the 169 ORFs annotated for the Merlin strain was estimated using GammaMap [42]. We assumed θ (neutral mutation rate per site), k (transitions/transversions ratio), and T (branch length) to vary among genes following log-normal distributions, whereas p (probability of adjacent codons to share the same selection coefficient) following a log-uniform distribution. For each gene we set the neutral frequencies of non-STOP codons (1/61). For selection coefficients we considered a uniform Dirichlet distribution with the same prior weight for each selection class. For each gene we run 100,000 iterations with thinning interval of 10 iterations.

### 3D structures

All crystallographic structures were derived from the Protein Data Bank (PDB) (see IDs in Figure legends). Images were rendered using PyMOL (The PyMOL Molecular Graphics System, Version 1.5.0.2 Schrödinger, LLC).

### Cell lines and HCMV strain

Primary human foreskin fibroblasts (HFFs, ATCC SCRC-1041, male), human retinal pigment epithelial cells (ARPE-19, ATCC CRL-2302, male), human epitelial adenocarcinoma cells (HeLa, ATCC CCL-2, female) and human embryonic kidney cells (HEK-293, ATCC CRL-1573, female) were cultured in Dulbecco’s Modified Eagle’s Medium (DMEM) supplemented with 10% Fetal Bovine Serum (FBS, Euroclone, Milano, Italy), 2 mM L-glutamine and 100 U/ml penicillin/streptomycin (Invitrogen, Carlsbad, CA, USA, Thermo Fisher Scientific, Waltham, MA, USA). All cell lines were grown at 37°C in a humidified 5% CO2 incubator.

HCMV strain TB40-BAC4 was propagated and titrated on HFFs by standard plaque assay [105, 106].

### Plasmids and Antibodies

pEPkan-S template plasmid (Addgene plasmid # 41017) [107] was used for the mutagenesis of vUL70. pSGIE86 IE1-encoding plasmid was used in quantitative nucleic acid analysis [108].

pCMV6-UL144 (gtA) (VC101305) was purchased from Origene. The sequence of UL144 (gtB) was synthesized and cloned in pCMV6-Entry vector by Origene custom service.

To study the timing of SP removal, the 24 bp DDK tag sequence (also referred to as FLAG) was inserted downstream the SP by a PCR site-direct mutagenesis approach. Briefly, for each construct (gtA and gtB), a pair of complementary primers were designed between the SP and the next sequence portion, and the DDK tag sequence was inserted right in the middle of both complementary primers at the end of SP sequence (S4 Table and Fig 8A). PCR was carried out in a 50 μl reaction containing 10 ng of specific plasmid template, 10 μM of each of the two primer pairs, 200 μM dNTPs, and 3 units of Pfu DNA polymerase (Promega). Reactions were performed under the following cycling conditions: initial denaturation at 95°C for 2 min; 35 cycles at 95°C for 30 s, 70°C for 30 s, and 72°C for 10 min; and a final extension at 72°C for 15 min. The PCR products were treated with 5 units of *Dpn*I (New England BioLabs) at 37°C for 2 hours and 5 μl aliquot of each digested PCR products was transformed into *E*. *coli* DH5α competent cells (ThermoFisher Scientific) by heat shock. The transformed cells were spread on a Luria-Bertani (LB) plate containing kanamycin antibiotic and incubated at 37°C over night. Colonies from each plate were grown and the plasmid DNA was isolated using Qiagen miniprep kits (Qiagen). To verify the correctness of the constructs, clones were sequenced using specific primers on pCMV6 backbone (S4 Table).

Mouse monoclonal anti-DDK–Clone 4C5 (TA50011-100, Origene) was used to detected DDK-tagged UL144. Rabbit antibodies against Sodium Potassium ATPase (ab76020), LAMP1 (ab24170), and SEC61a (ab221288) were purchased from Abcam. Goat polyclonal anti-EEA1 (N-19, sc-6415) was purchased from Santa Cruz Biotechnology. Rabbit polyclonal anti-calreticulin (PA3-900) was purchased from Thermo Fisher Scientific.

For the evaluation of the timing of SPs removal, rabbit anti-DDK polyclonal antibody (TA100023, Origene) and mouse monoclonal ANTI-FLAG M1 antibody (F3040, Merck) were used to detect the DDK-tag (irrespective of context) and the free N-terminus DDK-tag, respectively.

Alexa Fluor secondary antibodies Goat anti-Mouse IgG (H+L) - 488 (A-11001), Goat anti-Rabbit IgG (H+L) - 546 (A-11010), Donkey anti-Mouse IgG (H+L) - 488 (A-21202), Donkey anti-Rabbit IgG (H+L) - 546 (A-10040), and Donkey anti-Goat IgG (H+L) - 647 (A-21447) were purchased from Thermo Fisher Scientific.

### HCMV mutagenesis

Viruses used in this study were all bacterial artificial chromosome (BAC) clones. The clones of the endotheliotropic HCMV strain TB40-BAC4 (wild-type) viruses were generated using a markerless two-step RED-GAM recombination protocol [107, 109]. RED recombination was used to modify the TB40-BAC4 BAC that is derived from the endotheliotropic HCMV strain TB40/E [110]. The BAC of mutant virus vUL70 was generated with specific primers listed in S4 Table. Primers contained sequences of homology upstream and downstream the sites to be mutated, the mutation (lowercase), and sequences homologous to the pEPkan-S template plasmid (underlined) (Addgene plasmid # 41017; http://n2t.net/addgene:41017; RRID:Addgene_41017) [107]. All generated recombinant BAC DNAs were controlled for integrity and correctness by restriction length polymorphism and sequencing of the mutated region. HFF cells were used for reconstitution of recombinant viruses and virus stock production. The reconstitution from BAC DNA was performed as previously described [111].

### Focus Expansion Assay (FEA)

To analyze BAC clone transmission and spreading on different cell types, focus expansion assay (FEA) was performed as previously described [36]. Serial dilutions of HFFs (104 to 1) infected by the indicated viruses were co-cultured with an excess of uninfected HFFs or ARPE-19 cells in a 96 well plate. After 5 days of cocultivation, cells were fixed with cold methanol and HCMV immediate early antigen (CMV IE1/2 Monoclonal Antibody–CH160, Vyrusis Corporation) was detected by indirect immunoperoxidase staining (VECTASTAIN Universal Quick HRP Kit, R.T.U., Vector Laboratories). Stained slides were read with a Leica ICC50 HD microscope (Leica Microsystems). Infectious foci were defined as clusters of three or more antigen-positive cells. The number of infected cells in ten representative foci were counted. Plaque area and IEA positive nuclei were calculated using ImageJ software.

Statistical tests were performed using GraphPad Prism version 5.00 for Windows (GraphPad Software, San Diego California USA). One-way ANOVA followed by Tukey's post-hoc test was used for comparison of mutant viruses versus the wild-type.

### Growth competition assay

HFFs were plated in a p24 well and infected with 0.0005 PFU/ml of wild-type and vUL70 G294L each, wild-type and vUL70 R465K each, wild-type and vUL70 dm each. Virus growth was maintained for 28 days, while cells were split and fresh culture media replenished twice a week. At different time points, cells were collected and total genomic DNA was extracted using TRI Reagent solution (Sigma-Aldrich), according to the manufacture’s protocol, and eluted into 30 uL of water. Each head-to-head competition consisted of 2 biological replicates. The relative proportion of wild-type and mutant viruses was assessed using deep sequencing.

Library preparation for Illumina sequencing on NextSeq 550 platform was performed in accordance with Illumina tech note 15044223 Rev-B, using PCRBIO HS VeriFi Mix (PCR Biosystems), with two primer sets built to amplify PCR fragments around the 294 and 465 variants (S4 Table).

After purification with Ampure XP (Beckman Genomics), samples were quantified using Qubit high sensitivity reagents (ThermoFisher Scientific) and then pooled. Final pools of up to 96 libraries were quantified using high sensitivity Bioanalyzer reagents (Agilent) prior to sequencing 2X150 bp long paired end reads of the amplicons with NextSeq. Samples were supplemented with 1% phiX control library (Illumina). All reagents were used according to manufacturer’s recommendations.

The generated reads were processed to remove adaptors and low quality bases (q< 30). The bwa aligner [112] was used to map the selected reads to the *UL70* gene. The aligned sequences were analyzed with samtools [113] and homemade R-scripts to count the number of reads deriving from wild-type or mutant viruses. The statistical significance of an increasing trend in mutant read counts across time points were assessed with Cochran Armitage tests for trend. These were performed by considering the counts of sequence reads corresponding to the wild-type and the mutant virus as the response variable and time points as the explanatory variable with ordered levels.

Relative fitness (*f*) was calculated by applying the following formula:

$$f=\frac{1}{t}\cdot ln\frac{p(t)\cdot q(0)}{q(t)\cdot p(0)}$$

where *t* is time (expressed in days), *p* and *q* are the proportion of reads deriving from mutants and wild-type viruses, respectively [114, 115].

### Immunofluorescence and confocal microscopy

HeLa/HEK-293 cells were seeded (0.3 x 10^5^ cells/well) onto coverslips treated with 0.1 ug/mL poly-L-lysine. Transient transfections were performed using Lipofectamine 2000 (Thermo Fisher Scientific, 11,668,027) with 2.5 μg of plasmid DNA (pCMV6-UL144 gtA and pCMV6-UL144 gtB). At 3, 4, 5, 6, 9 and 24 hours after transfection, cells were fixed with 4% paraformaldehyde (Santa Cruz Biotechnology, sc-281692) and permeabilized with phosphate-buffered saline (PBS; Euroclone, ECB4053L) containing 0.1% saponin (Sigma-Aldrich, S4521) and 1% bovine serum albumin (Sigma-Aldrich, A9647).

Cells were then incubated in the same buffer for 2h with primary Abs (1:50) and revealed using the secondary Abs Alexa Fluor 488, 546 and 647 (1:500).

Confocal microscopy was performed with a Yokogawa CSU-X1 spinning disk confocal on a Nikon Ti-E inverted microscope equipped with a Nikon 60x/1.40 oil Plan Apochromat objective and were acquired with an Andor Technology iXon3 DU-897-BV EMCCD camera (Nikon Instruments S.p.A., Firenze, Italy). The investigator was blinded as to the nature of the sample analyzed. Pearson correlation coefficients for protein co-localization were determined with Fiji ImageJ software using the COLOC2 plugin.

Statistical analyses were performed using GraphPad Prism version 8.1.1 for Windows (GraphPad Software, San Diego, California USA, www.graphpad.com).

Significance was calculated with two-way ANOVA followed by Sidak’s multiple comparisons test or with Student’s t test for unpaired variables (two-tailed), as detailed in the legend of the figures (Figs 6, 7 and 8). Pearson’s correlation coefficients are reported as mean ± standard error of the mean (SEM); n represents individual data. For the statistical analysis, a Fisher transformation in z-scores of Pearson’s correlation coefficients was performed. p values of less than 0.05 were considered significant.

## Supporting information

S1 Fig Phylogenetic relationships for the *US6* and *US12* gene families. Protein sequences of family homologs were searched for as described in the Materials and Methods. Phylogenetic trees were constructed using RAxML with 1000 bootstrap replicates (posterior probabilities are reported at nodes). Orthologous gene groups, shown in red on the tree and denoted by the gray shading, were inferred on the basis of the tree topology and of bootstrap values > 70. Magenta asterisks denote genes that are frequently deleted/mutated in clinical isolates [16].
(PDF)

S2 Fig Analysis of selective patterns for catarrhini-infecting CMVs. The dN/dS parameter is compared among genes showing different function **(A)** or by the location of the encoded protein **(B)**. p values derive from Wilcoxon Rank-Sum tests with FDR correction. Star indicates significant p value (< 0.05).
(PDF)

S3 Fig Selective patterns in HCMV clinical isolates from different compartments. Distribution of selection coefficients (*γ*) for all coding genes of HCMV clinical isolates sampled from amniotic fluid, urine, and blood/plasma. Selection coefficients were calculated for all codons and genes were grouped on the basis of the results of the branch-site analysis.
(PDF)

S4 Fig Population genetics-phylogenetics analysis of HCMV genes. Violin plots (median, white dot; interquartile range, black bar) of selection coefficients for genes of HCMV isolates deriving from the blood/plasma (red), urine (yellow), and amniotic fluid (light blue). Selection coefficients (γ) are classified as strongly beneficial (100, 50), moderately beneficial (10, 5), weakly beneficial (1), neutral (0), weakly deleterious (−1), moderately deleterious (−5, −10), strongly deleterious (−50, −100), and inviable (−500). The gray shading denotes different degrees of constraint based on selection coefficients.
(PDF)

S5 Fig Positively selected sites detected by gammaMap analysis. Positively selected sites (black arrows) were mapped onto the topological domains of HCMV proteins. Protein domain information was obtained from the Uniprot and SMART databases. Positions refer to proteins of the Merlin strain (NC_006273) (see also S8 Table).
(PDF)

S6 Fig Positive selection, sequence diversity, and ADs in envelope glycoproteins. Protein domain information and positively selected sites of gB, gH, gM and gO are as in S5 Fig. Plots below the schematic representations of protein domains report the number of amino acidic substitutions per site, as inferred from the sequences used for gammaMap analysis (see S7 Table) and provided as a measure of polymorphism. Black and blue triangles denote sites identified with gammaMap or with the branch-site test, respectively. Grey boxes indicated linear epitopes mapped onto ADs. The name of major neutralizing antibodies targeting these epitopes are reported. gB and gH present some region with high sequence polymorphism. They are also highly immunogenic and represent major targets of neutralizing antibodies (see text). gM displays very low levels of sequence diversity among strains, whereas high sequence divergence was observed in gO, especially at the N-terminus, coincident with the signals of positive selection. For gO, no AD/epitope has been identified yet.
(PDF)

S7 Fig Analysis of UL144 gtA and gtB localization in HEK-293 cells. HEK-293 cells were transfected with pCMV6-UL144 gtA and pCMV6-UL144 gtB. **(A)** Twenty-four hours after transfection cells were fixed and immunostained with antibodies against the DDK tag (green) and the plasma membrane protein sodium potassium ATPase (red). Nuclei were counterstained with DAPI. Arrows indicates co-localization at the plasma membrane. Scale bar: 10 μm. **(B)** Twenty-four hours after transfection cells were fixed and immunostained with antibodies against the DDK tag (green), the lysosomal marker LAMP1 (red), and the early endosomal marker EEA1 (blue). Co-localization of DDK with LAMP1 (yellow) or EEA1 (light blue) is showed in the merge images. The small panels show a higher magnification of the area indicated in the squares. Scale bar: 10 μm. Pearson’s correlation coefficients for DDK/LAMP1 or DDK/EEA1 co-localization were reported in the graphs as mean ± SEM (*t* test; n > 30). Scale bar: 10 μm. **(C)** Four hours after transfection cells were fixed and immunostained with antibodies against the DDK tag (green) and Sec61A (red). Nuclei were counterstained with DAPI. Yellow in the merge images indicates co-localization. Pearson’s correlation coefficients for DDK/Sec61A co-localization were reported in the graphs as mean ± SEM (*t* test; n > 25). Scale bar: 10 μm. **(D)** Six hours after transfection cells were fixed and immunostained with antibodies against the DDK tag (green) and calreticulin (red). Nuclei were counterstained with DAPI. Yellow in the merge images indicates co-localization. Pearson’s correlation coefficients for DDK/Calreticulin co-localization were reported in the graphs as mean ± SEM (*t* test; n > 25). Scale bar: 10 μm.
(PDF)

S1 Table List of sequences used for the branch-site test. (PDF)

S2 Table CMV genes excluded from the branch-site test. (PDF)

S3 Table Likelihood ratio test (LRT) statistics for models of variable selective pressure on the HCMV branch. (PDF)

S4 Table List of primers. (PDF)

S5 Table Focus expansion assay (FEA). (PDF)

S6 Table List of HCMV strains used for HCMV ancestral outgroup reconstruction. (PDF)

S7 Table List of HCMV strains used for gammaMap analyses. (PDF)

S8 Table List of positively selected sites detected with gammaMap. (XLSX)
