# Supplementary material for: Past and ongoing adaptation of human cytomegalovirus to its host
Source: PLoS Pathog. 2020 May 8;16(5):e1008476. doi: 10.1371/journal.ppat.1008476 (PMC7239485; doi:10.1371/journal.ppat.1008476)
Supplement: S1 Fig — Protein sequences of family homologs were searched for as described in the Materials and Methods. Phylogenetic trees were constructed using RAxML with 1000 bootstrap replicates (posterior probabilities are reported at nodes). Orthologous gene groups, shown in red on the tree and denoted by the gray shading, were inferred on the basis of the tree topology and of bootstrap values > 70. Magenta asterisks denote genes that are frequently deleted/mutated in clinical isolates [16]. (PDF) [file ppat.1008476.s001.pdf]

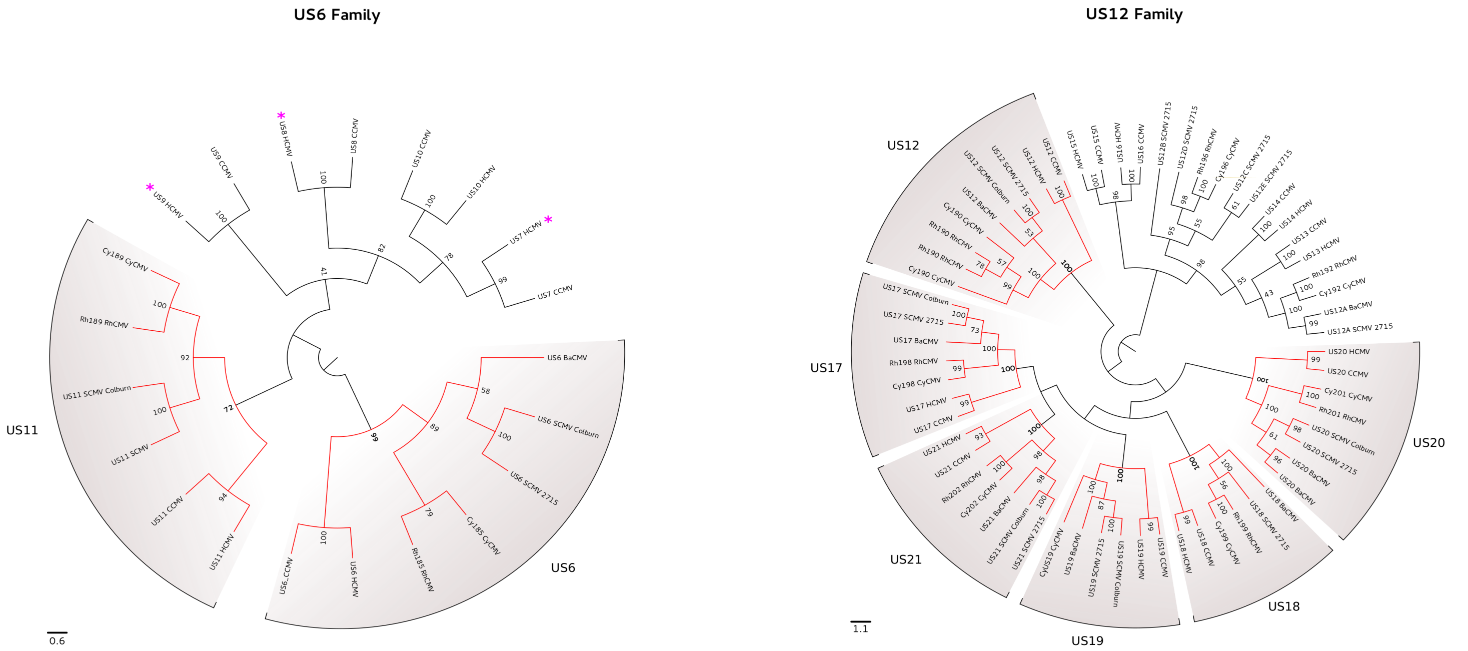

S1 Fig. Phylogenetic relationships for the US6 and US12 gene families. Protein sequences of family homologs were searched for as described in the Materials and Methods. Phylogenetic trees were constructed using RAxML with 1000 bootstrap replicates (posterior probabilities are reported at nodes). Orthologous gene groups, shown in red on the tree and denoted by the gray shading, were inferred on the basis of the tree topology and of bootstrap values >70. Magenta asterisks denote genes that are frequently deleted/mutated in clinical isolates [16].
