# Supplementary figures and images for: Past and ongoing adaptation of human cytomegalovirus to its host
Source: PLoS Pathog. 2020 May 8;16(5):e1008476. doi: 10.1371/journal.ppat.1008476 (PMC7239485; doi:10.1371/journal.ppat.1008476)

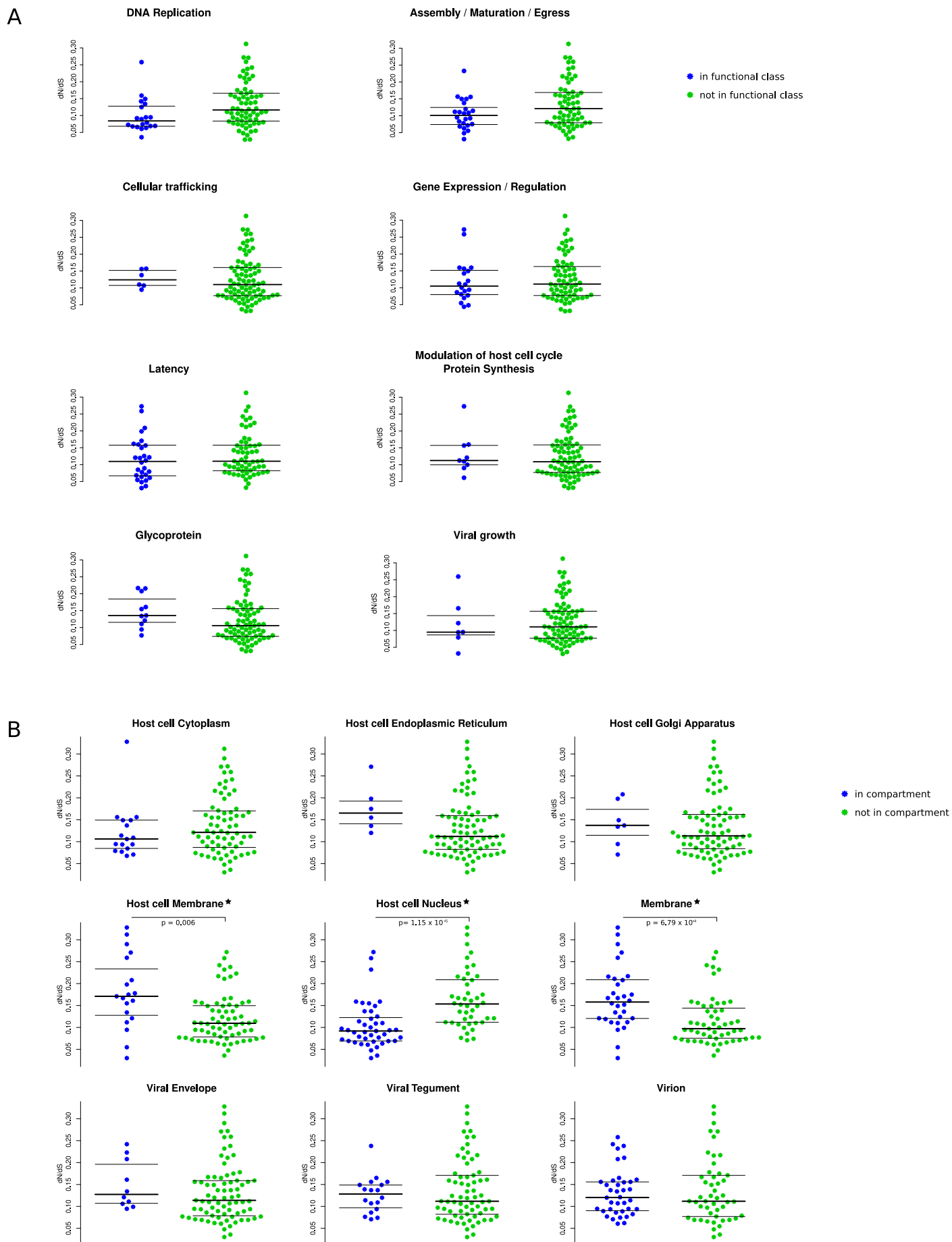

Supplement: S2 Fig — The dN/dS parameter is compared among genes showing different function (A) or by the location of the encoded protein (B). p values derive from Wilcoxon Rank-Sum tests with FDR correction. Star indicates significant p value (< 0.05). (PDF) [file ppat.1008476.s002.pdf]
