# Supplementary material for: Past and ongoing adaptation of human cytomegalovirus to its host
Source: PLoS Pathog. 2020 May 8;16(5):e1008476. doi: 10.1371/journal.ppat.1008476 (PMC7239485; doi:10.1371/journal.ppat.1008476)
Supplement: S3 Fig — Distribution of selection coefficients (γ) for all coding genes of HCMV clinical isolates sampled from amniotic fluid, urine, and blood/plasma. Selection coefficients were calculated for all codons and genes were grouped on the basis of the results of the branch-site analysis. (PDF) [file ppat.1008476.s003.pdf]

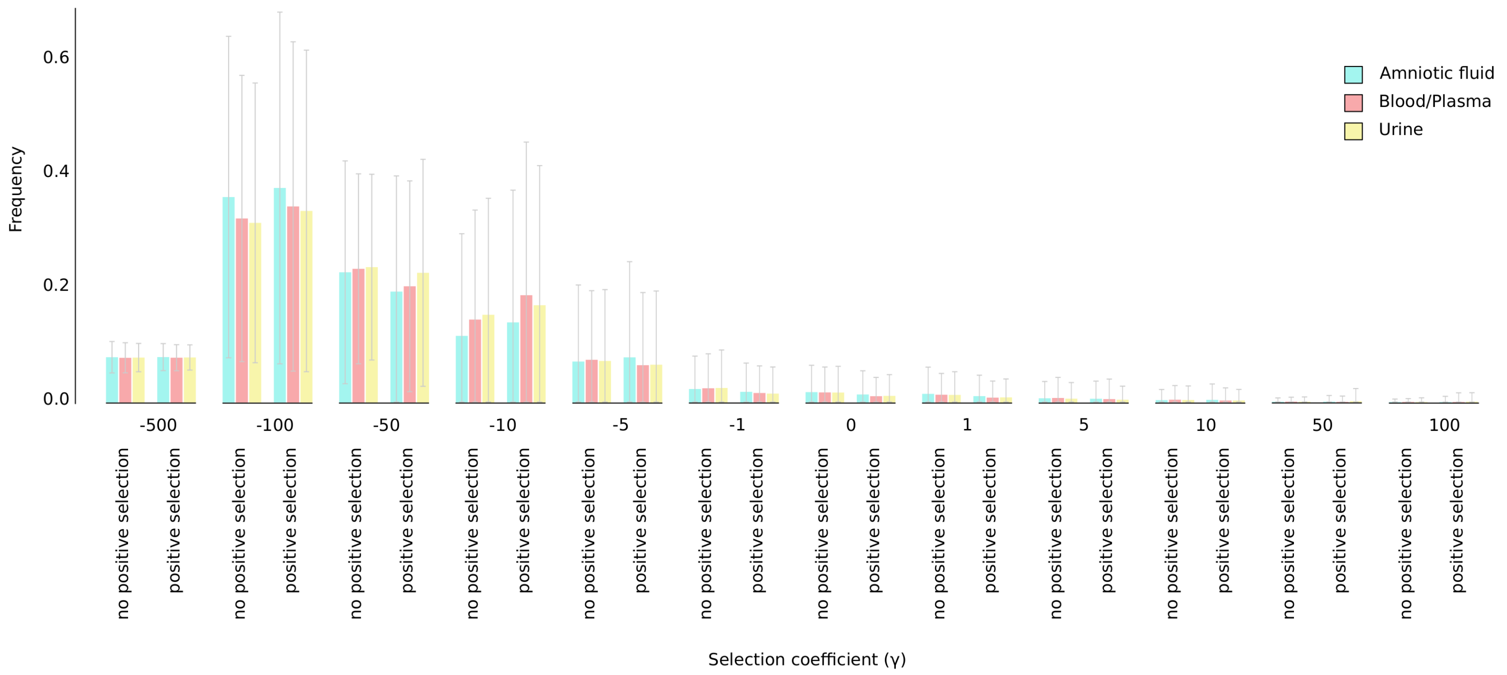

S3 Fig. Selective patterns in HCMV clinical isolates from different compartments. Distribution of selection coefficients (γ) for all coding genes of HCMV clinical isolates sampled from amniotic fluid, urine, and blood/plasma. Selection coefficients were calculated for all codons and genes were grouped on the basis of the results of the branch-site analysis.
