# Supplementary material for: Past and ongoing adaptation of human cytomegalovirus to its host
Source: PLoS Pathog. 2020 May 8;16(5):e1008476. doi: 10.1371/journal.ppat.1008476 (PMC7239485; doi:10.1371/journal.ppat.1008476)
Supplement: S4 Fig — Violin plots (median, white dot; interquartile range, black bar) of selection coefficients for genes of HCMV isolates deriving from the blood/plasma (red), urine (yellow), and amniotic fluid (light blue). Selection coefficients (γ) are classified as strongly beneficial (100, 50), moderately beneficial (10, 5), weakly beneficial (1), neutral (0), weakly deleterious (−1), moderately deleterious (−5, −10), strongly deleterious (−50, −100), and inviable (−500). The gray shading denotes different degrees of constraint based on selection coefficients. (PDF) [file ppat.1008476.s004.pdf]

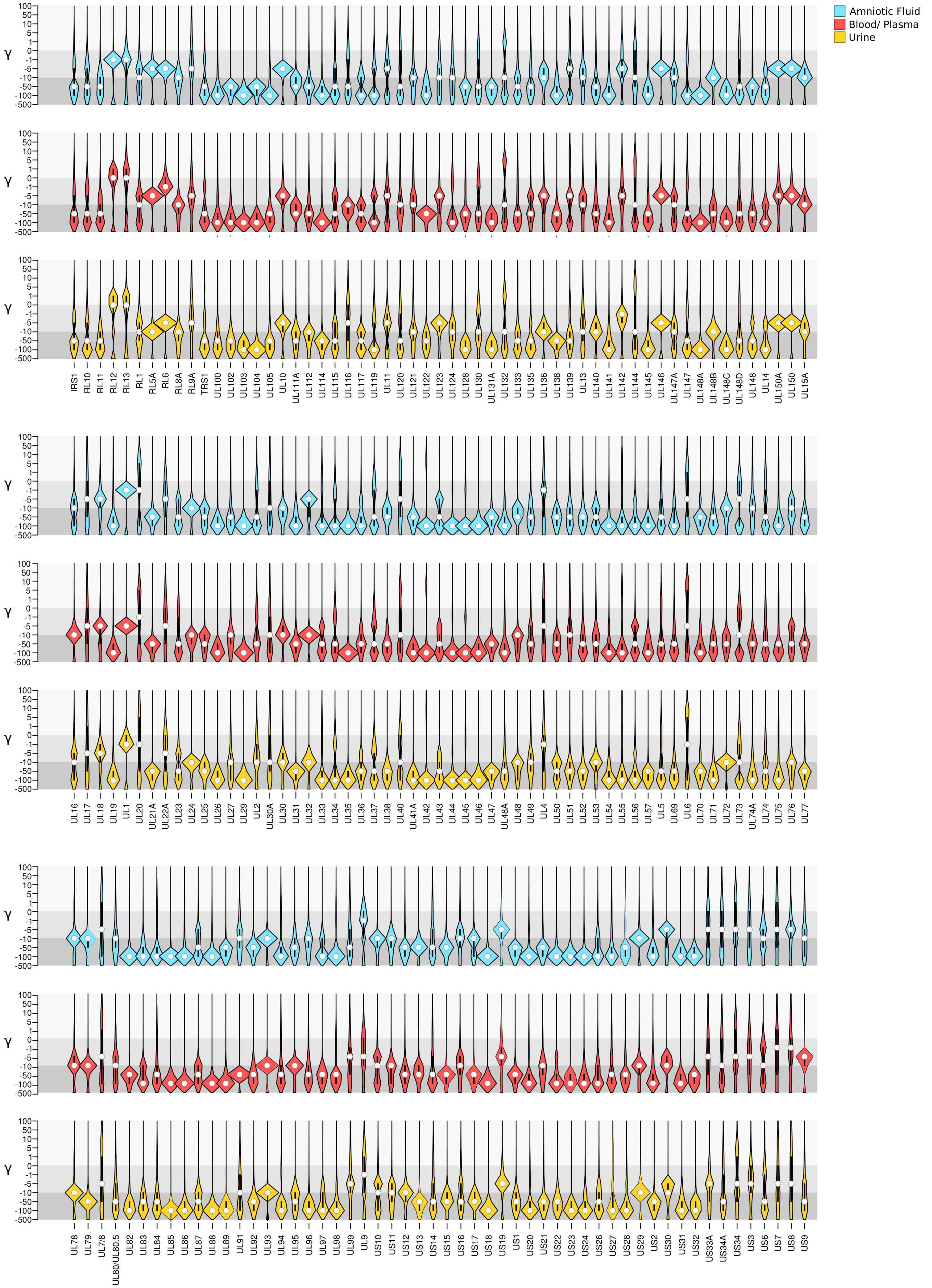

S4 Fig. Population genetics-phylogenetics analysis of HCMV genes. Violin plots (median, white dot; interquartile range, black bar) of selection coefficients for genes of HCMV isolates deriving from the blood/plasma (red), urine (yellow), and amniotic fluid (light blue). Selection coefficients (γ) are classified as strongly beneficial (100, 50), moderately beneficial (10, 5), weakly beneficial (1), neutral (0), weakly deleterious (−1), moderately deleterious (−5, −10), strongly deleterious (−50, −100), and inviable (−500). The gray shading denotes different degrees of constraint based on selection coefficients.
