# Supplementary material for: Past and ongoing adaptation of human cytomegalovirus to its host
Source: PLoS Pathog. 2020 May 8;16(5):e1008476. doi: 10.1371/journal.ppat.1008476 (PMC7239485; doi:10.1371/journal.ppat.1008476)
Supplement: S5 Fig — Positively selected sites (black arrows) were mapped onto the topological domains of HCMV proteins. Protein domain information was obtained from the Uniprot and SMART databases. Positions refer to proteins of the Merlin strain (NC_006273) (see also S8 Table). (PDF) [file ppat.1008476.s005.pdf]

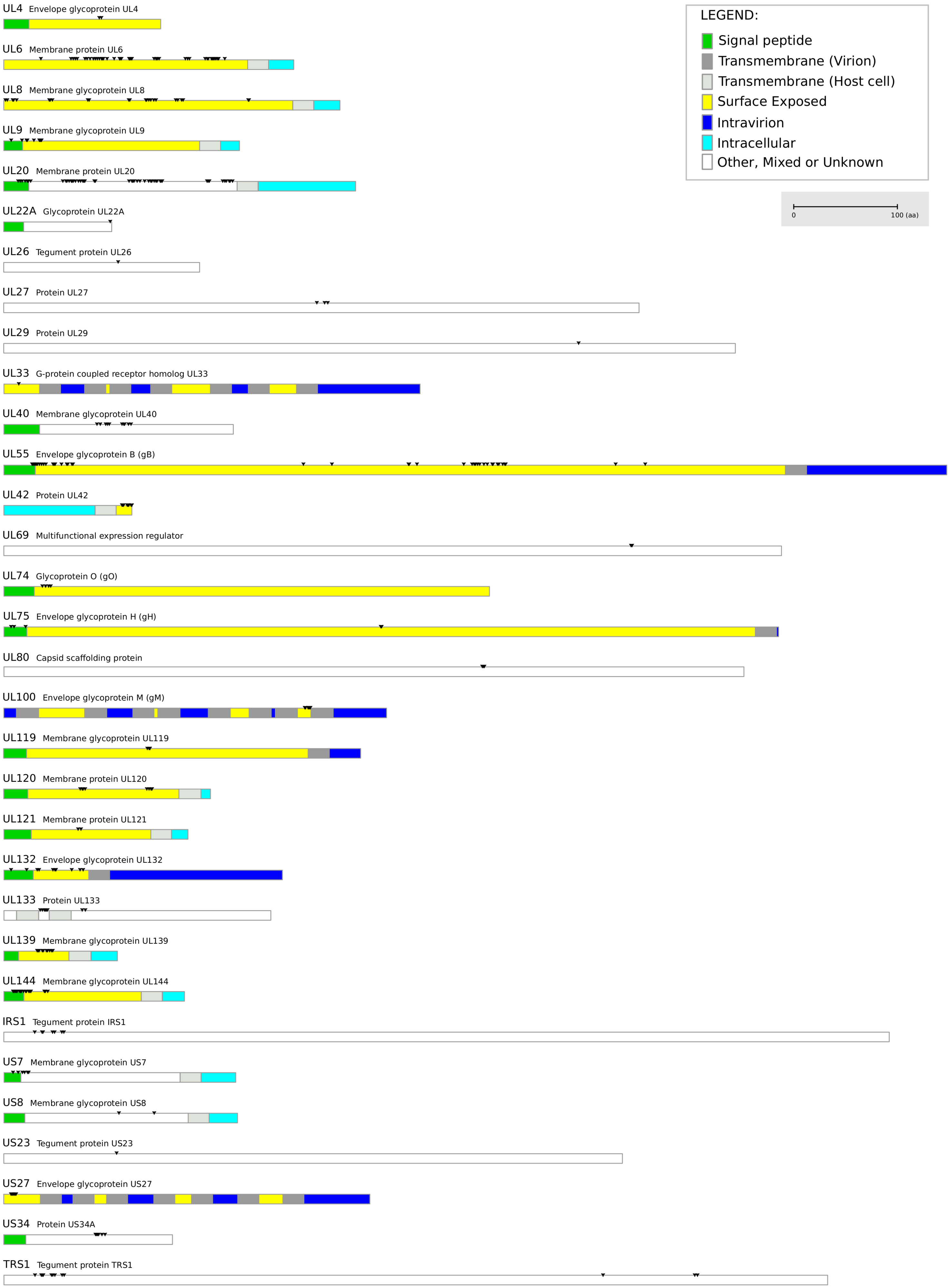

S5 Fig. Positively selected sites detected by gammaMap analysis. Positively selected sites (black arrows) were mapped onto the topological domains of HCMV proteins. Protein domain information was obtained from the Uniprot and SMART databases. Positions refer to proteins of the Merlin strain (NC_006273) (see also S8 Table).
