# Supplementary material for: Past and ongoing adaptation of human cytomegalovirus to its host
Source: PLoS Pathog. 2020 May 8;16(5):e1008476. doi: 10.1371/journal.ppat.1008476 (PMC7239485; doi:10.1371/journal.ppat.1008476)
Supplement: S6 Fig — Protein domain information and positively selected sites of gB, gH, gM and gO are as in S5 Fig. Plots below the schematic representations of protein domains report the number of amino acidic substitutions per site, as inferred from the sequences used for gammaMap analysis (see S7 Table) and provided as a measure of polymorphism. Black and blue triangles denote sites identified with gammaMap or with the branch-site test, respectively. Grey boxes indicated linear epitopes mapped onto ADs. The name of major neutralizing antibodies targeting these epitopes are reported. gB and gH present some region with high sequence polymorphism. They are also highly immunogenic and represent major targets of neutralizing antibodies (see text). gM displays very low levels of sequence diversity among strains, whereas high sequence divergence was observed in gO, especially at the N-terminus, coincident with the signals of positive selection. For gO, no AD/epitope has been identified yet. (PDF) [file ppat.1008476.s006.pdf]

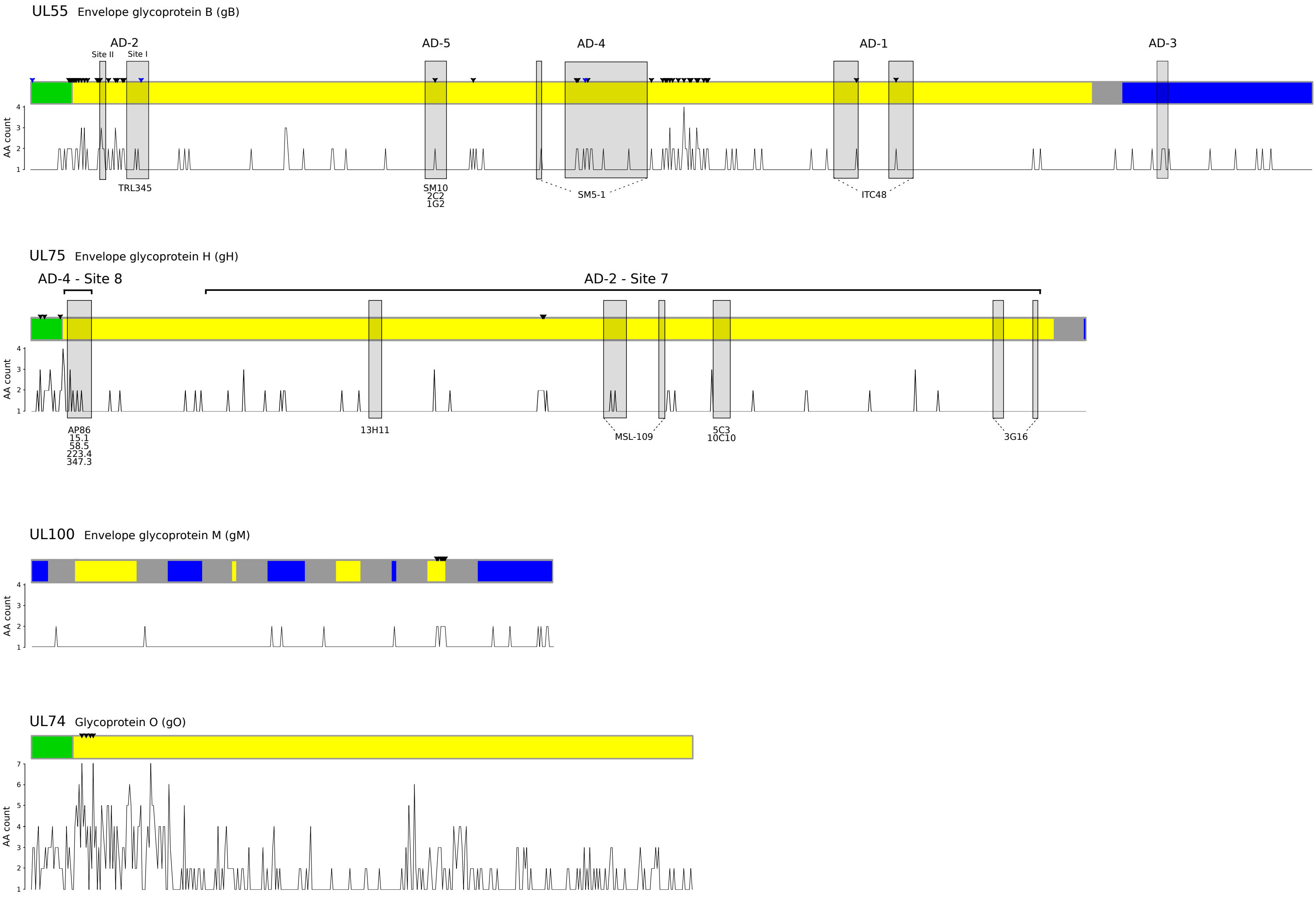

S6 Fig. Positive selection, sequence diversity, and ADs in envelope glycoproteins. Protein domain information and positively selected sites of gB, gH, gM and gO are as in S5 Fig. Plots below the schematic representations of protein domains report the number of amino acids per site, as inferred from the sequences used for gammaMap analysis (see S7 Table) and provided as a measure of polymorphism. Black and blue triangles denote sites identified with gammaMap or with the branch-site test, respectively. Grey boxes indicated linear epitopes mapped onto ADs. The name of major neutralizing antibodies targeting these epitopes are reported. gB and gH present some region with high sequence polymorphism. They are also highly immunogenic and represent major targets of neutralizing antibodies (see text). gM displays very low levels of sequence diversity among strains, whereas high sequence divergence was observed in gO, especially at the N-terminus, coincident with the signals of positive selection. For gO, no AD/epitope has been identified yet**.**
