# Supplementary material for: Past and ongoing adaptation of human cytomegalovirus to its host
Source: PLoS Pathog. 2020 May 8;16(5):e1008476. doi: 10.1371/journal.ppat.1008476 (PMC7239485; doi:10.1371/journal.ppat.1008476)
Supplement: S7 Fig — HEK-293 cells were transfected with pCMV6-UL144 gtA and pCMV6-UL144 gtB. (A) Twenty-four hours after transfection cells were fixed and immunostained with antibodies against the DDK tag (green) and the plasma membrane protein sodium potassium ATPase (red). Nuclei were counterstained with DAPI. Arrows indicates co-localization at the plasma membrane. Scale bar: 10 μm. (B) Twenty-four hours after transfection cells were fixed and immunostained with antibodies against the DDK tag (green), the lysosomal marker LAMP1 (red), and the early endosomal marker EEA1 (blue). Co-localization of DDK with LAMP1 (yellow) or EEA1 (light blue) is showed in the merge images. The small panels show a higher magnification of the area indicated in the squares. Scale bar: 10 μm. Pearson’s correlation coefficients for DDK/LAMP1 or DDK/EEA1 co-localization were reported in the graphs as mean ± SEM (t test; n > 30). Scale bar: 10 μm. (C) Four hours after transfection cells were fixed and immunostained with antibodies against the DDK tag (green) and Sec61A (red). Nuclei were counterstained with DAPI. Yellow in the merge images indicates co-localization. Pearson’s correlation coefficients for DDK/Sec61A co-localization were reported in the graphs as mean ± SEM (t test; n > 25). Scale bar: 10 μm. (D) Six hours after transfection cells were fixed and immunostained with antibodies against the DDK tag (green) and calreticulin (red). Nuclei were counterstained with DAPI. Yellow in the merge images indicates co-localization. Pearson’s correlation coefficients for DDK/Calreticulin co-localization were reported in the graphs as mean ± SEM (t test; n > 25). Scale bar: 10 μm. (PDF) [file ppat.1008476.s007.pdf]

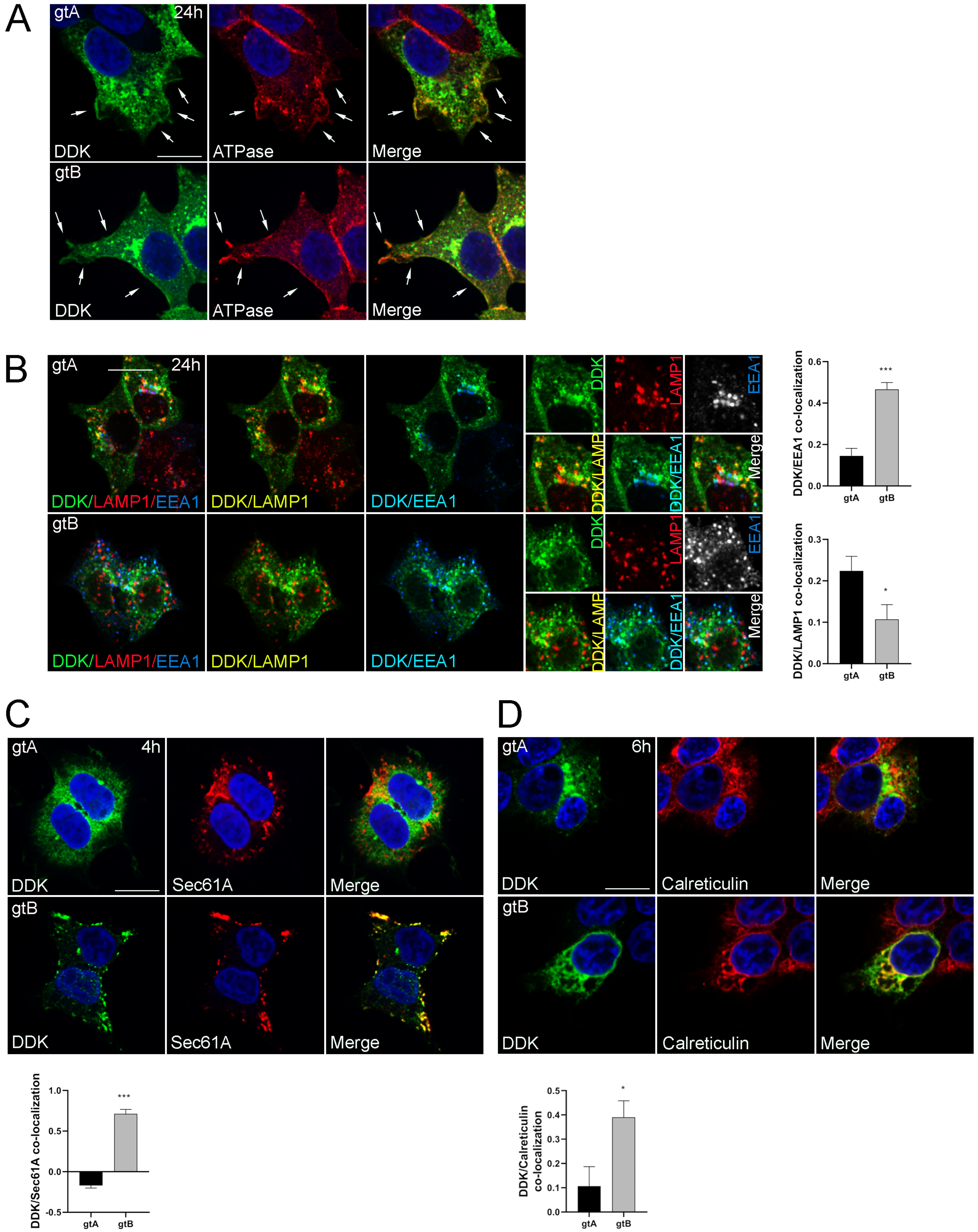

S7 Fig. Analysis of UL144 gtA and gtB localization in HEK-293 cells. HEK-293 cells were transfected with pCMV6-UL144 gtA and pCMV6-UL144 gtB. **(A)** Twenty-four hours after transfection cells were fixed and immunostained with antibodies against the DDK tag (green) and the plasma membrane protein sodium potassium ATPase (red). Nuclei were counterstained with DAPI. Arrows indicates co-localization at the plasma membrane. Scale bar: 10 µm. **(B)** Twenty-four hours after transfection cells were fixed and immunostained with antibodies against the DDK tag (green), the lysosomal marker LAMP1 (red), and the early endosomal marker EEA1 (blue). Co-localization of DDK with LAMP1 (yellow) or EEA1 (light blue) is showed in the merge images. The small panels show a higher magnification of the area indicated in the squares. Scale bar: 10 µm. Pearson's correlation coefficients for DDK/LAMP1 or DDK/EEA1 co-localization were reported in the graphs as mean ± SEM (t test; n>30). Scale bar: 10 µm. **(C)** Four hours after transfection cells were fixed and immunostained with antibodies against the DDK tag (green) and Sec61A (red). Nuclei were counterstained with DAPI. Yellow in the merge images indicates co-localization. Pearson's correlation coefficients for DDK/Sec61A co-localization were reported in the graphs as mean ± SEM (t test; n>25). Scale bar: 10 µm. **(D)** Six hours after transfection cells were fixed and immunostained with antibodies against the DDK tag (green) and calreticulin (red). Nuclei were counterstained with DAPI. Yellow in the merge images indicates co-localization. Pearson's correlation coefficients for DDK/Calreticulin co-localization were reported in the graphs as mean ± SEM (t test; n>25). Scale bar: 10 µm.
