# Supplementary material for: Past and ongoing adaptation of human cytomegalovirus to its host
Source: PLoS Pathog. 2020 May 8;16(5):e1008476. doi: 10.1371/journal.ppat.1008476 (PMC7239485; doi:10.1371/journal.ppat.1008476)
Supplement: S1 Table — (PDF) [file ppat.1008476.s008.pdf]

S1 Table . List of sequences used for the branch-site test.

Non-human primate CMVs					
**Strain Name**	**Accession ID**	**Virus name (abbreviation)**	**Virus Species**	**Host**	**Country**
CCMV Heberling *	NC_003521	Chimpanzee cytomegalovirus (CCMV Heberling)	*Panine betaherpesvirus 2*	*Pan troglodytes*	USA
DrCMV OCOM6-2 *	KR297253	Drill monkey cytomegalovirus (DrCMV OCOM6-2)	*Mandrilline betaherpesvirus 1*	*Mandrillus leucophaeus*	Nigeria
BaCMV OCOM4-52 *	KR351281	Chacma baboon cytomegalovirus (BaCMV OCOM4-52)	*Papiine betaherpesvirus 4*	*Papio ursinus*	South Africa
SCMV 2715 *	NC_012783	Simian cytomegalovirus (SCMV 2715)	*Cercopithecine herpesvirus 5*	*Cercopithecus aethiops*	USA
SCMV Colburn *	FJ483969	Simian cytomegalovirus (SCMV Colburn)	*Cercopithecine herpesvirus 5*	*Homo sapiens*	USA
CyCMV 31906	KX689263	Cynomolgus macaque cytomegalovirus (CyCMV 31906)	*Macacine betaherpesvirus 3*	*Macaca fascicularis*	USA
CyCMV 31907	KX689264	Cynomolgus macaque cytomegalovirus (CyCMV 31907)	*Macacine betaherpesvirus 3*	*Macaca fascicularis*	USA
CyCMV 31908 *	NC_033176	Cynomolgus macaque cytomegalovirus (CyCMV 31908)	*Macacine betaherpesvirus 3*	*Macaca fascicularis*	USA
CyCMV 31909	KX689266	Cynomolgus macaque cytomegalovirus (CyCMV 31909)	*Macacine betaherpesvirus 3*	*Macaca fascicularis*	USA
CyCMV Mauritius	KP796148	Cynomolgus macaque cytomegalovirus (CyCMV Mauritius)	*Macacine betaherpesvirus 3*	*Macaca fascicularis*	Canada
CyCMV Ottawa *	JN227533	Cynomolgus macaque cytomegalovirus (CyCMV Ottawa)	*Macacine betaherpesvirus 3*	*Macaca fascicularis*	Canada
RhCMV 19262	KX689267	Rhesus macaque cytomegalovirus (RhCMV 19262)	*Macacine betaherpesvirus 3*	*Macaca mulatta*	USA
RhCMV 19936 *	KX689268	Rhesus macaque cytomegalovirus (RhCMV 19936)	*Macacine betaherpesvirus 3*	*Macaca mulatta*	USA
RhCMV 24514	KX689269	Rhesus macaque cytomegalovirus (RhCMV 24514)	*Macacine betaherpesvirus 3*	*Macaca mulatta*	USA
RhCMV 68-1 *	NC_006150	Rhesus macaque cytomegalovirus (RhCMV 68-1)	*Macacine betaherpesvirus 3*	*Macaca mulatta*	USA
RhCMV CMV 180.92	DQ120516	Rhesus macaque cytomegalovirus (RhCMV 180.92)	*Macacine betaherpesvirus 3*	*Macaca mulatta*	-N/A-

HCMVs					
**Strain Name**	**Accession ID**	**Sample Type**	**Virus Species**	**Isolation year**	**Country**
PRA6	KY490068	Amniotic fluid	*Human betaherpesvirus 5*	2015	Czech Republic
NAN1LA	KU550087	Amniotic fluid	*Human betaherpesvirus 5*	2011	France
JER4041	KR534206	Amniotic fluid	*Human betaherpesvirus 5*	2005	Israel
JER4053	KR534207	Amniotic fluid	*Human betaherpesvirus 5*	2009	Israel
JER5268	KR534210	Amniotic fluid	*Human betaherpesvirus 5*	2012	Israel
AF1	GU179291	Amniotic fluid	*Human betaherpesvirus 5*	2003	Italy
PAV1	KJ361959	Amniotic fluid	*Human betaherpesvirus 5*	2005	Italy
UKNEQAS2	KT634296	Amniotic fluid	*Human betaherpesvirus 5*	2013	Australia
UK/Lon1/Blood/2013	KT726947	Blood	*Human betaherpesvirus 5*	2013	United Kingdom

JHC *	HQ380895	Blood	*Human betaherpesvirus 5*	2003	South Korea
HANRTR10	KY490078	Blood	*Human betaherpesvirus 5*	2010	Germany
HANSCTR13	KY490088	Blood	*Human betaherpesvirus 5*	2011	Germany
HANSCTR9	KY490083	Blood	*Human betaherpesvirus 5*	2016	Germany
HANChild4	KY123649	Bronchial secretions	*Human betaherpesvirus 5*	2012	Germany
VR1814 *	GU179289	Cervical secretions	*Human betaherpesvirus 5*	1996	Italy
HANRTR4	KY123651	Plasma	*Human betaherpesvirus 5*	2015	Germany
BE/21/2010	KC519322	Urine	*Human betaherpesvirus 5*	2010	Belgium
3301	GQ466044	Urine	*Human betaherpesvirus 5*	2001	United Kingdom
U11	GU179290	Urine	*Human betaherpesvirus 5*	2003	United Kingdom
PRA1	KY490063	Urine	*Human betaherpesvirus 5*	2006	Czech Republic
PRA5	KY490067	Urine	*Human betaherpesvirus 5*	2009	Czech Republic
PRA7	KY490069	Urine	*Human betaherpesvirus 5*	2010	Czech Republic
PRA8	KY490070	Urine	*Human betaherpesvirus 5*	2012	Czech Republic
NANU	KU550090	Urine	*Human betaherpesvirus 5*	2013	France
HANRTR6	KY490075	Vitreous body fluid	*Human betaherpesvirus 5*	2014	Germany

* Asterisks denote viruses that were included in the analysis of selective patterns of catarrhini-infecting CMVs
