# Supplementary material for: Past and ongoing adaptation of human cytomegalovirus to its host
Source: PLoS Pathog. 2020 May 8;16(5):e1008476. doi: 10.1371/journal.ppat.1008476 (PMC7239485; doi:10.1371/journal.ppat.1008476)
Supplement: S2 Table — (PDF) [file ppat.1008476.s009.pdf]

S2 Table. CMV genes excluded from the branch-site analysis**.**

Gene Name	Motivation
IRS1	Too few orthologs
TRS1	Too few orthologs
RL5A	Too few orthologs
RL6	Too few orthologs
RL8A	Too few orthologs
RL9A	Too few orthologs
RL10	Too few orthologs
RL11	Coding alignment too short
RL12	Too few orthologs
RL13	Too few orthologs
UL1	Too few orthologs
UL2	Too few orthologs
UL4	Too few orthologs
UL5	Too few orthologs
UL7	Coding alignment too short
UL8	Too few orthologs
UL9	Too few orthologs
UL10	Too few orthologs
UL11	Too few orthologs
UL15A	Too few orthologs
UL16	Too few orthologs
UL17	Coding alignment too short
UL18	Too few orthologs
UL19	Coding alignment too short
UL21A	Coding alignment too short
UL22A	Too few orthologs
UL30	Coding alignment too short
UL30A	Too few orthologs
UL40	Coding alignment too short
UL41A	Coding alignment too short
UL48A	Coding alignment too short
UL73	Coding alignment too short
UL74A	Coding alignment too short
UL83	Too few orthologs
UL91	Coding alignment too short
UL99	Coding alignment too short
UL121	Coding alignment too short
UL123 Reg1	Coding alignment too short
UL124	Coding alignment too short
UL133	Too few orthologs
UL135	Too few orthologs
UL136	Too few orthologs
UL138	Too few orthologs
UL139	Too few orthologs
UL140	Too few orthologs
UL142	Too few orthologs
UL146	Coding alignment too short
UL147A	Coding alignment too short
UL148A	Too few orthologs

UL148B	Too few orthologs
UL148C	Too few orthologs
UL148D	Too few orthologs
UL150	Too few orthologs
UL150A	Too few orthologs
US6	Coding alignment too short
US7	Too few orthologs
US8	Coding alignment too short
US9	Too few orthologs
US10	Too few orthologs
US13	Too few orthologs
US14	Too few orthologs
US15	Too few orthologs
US16	Too few orthologs
US27	Too few orthologs
US31	Coding alignment too short
US33A	Too few orthologs
US34	Too few orthologs
US34A	Too few orthologs
