# Supplementary material for: Past and ongoing adaptation of human cytomegalovirus to its host
Source: PLoS Pathog. 2020 May 8;16(5):e1008476. doi: 10.1371/journal.ppat.1008476 (PMC7239485; doi:10.1371/journal.ppat.1008476)
Supplement: S3 Table — (PDF) [file ppat.1008476.s010.pdf]

S3 Table. Likelihood ratio test (LRT) statistics for models of variable selective pressure on the HCMV branch.

Gene Name	Alignment Length (nt)	N° of strains	Tree Length[a]	-2ΔlnL[b]	MA vs MA1 *p*-value	MA vs MA1 FDR corrected *p*-value	MEME/BEB sites
RL1	390	36	18.683	0.13687	0.71141	0.90586	
UL6	462	37	7.063	3.57933	0.0585	0.12052	
UL13	375	31	6.569	0.05221	0.81926	0.99768	
UL14	693	40	7.512	4.2311	0.03969	0.09396	
UL20	525	40	9.540	5.87181	0.01539	**0.04957**	--
UL23	765	40	7.116	1.88841	0.16938	0.31691	
UL24	891	41	6.106	0.12999	0.71844	0.90586	
UL25	1395	41	7.972	14.3327	0.00015	**0.00227**	P68, N248, S372, I556, S770
UL26	516	41	6.080	4.62534	0.0315	0.08120	
UL27	1536	41	6.092	4.1836	0.04082	0.09470	
UL29 Reg1	1398	28	5.343	6.71660	0.00955	**0.03259**	A208
UL29 Reg2	579	28	3.813	0	1	1	
UL31	1347	40	5.582	9.93962	0.00162	**0.01118**	V481
UL32	1107	41	7.201	8.56468	0.00343	**0.01729**	--
UL33	1071	36	6.470	11.02253	0.0009	**0.00696**	N337
UL34	789	41	4.763	0.147056	0.70136	0.90398	
UL35	1491	39	7.140	18.82381	1.434×10^{-5}	**0.00083**	L167, R268, L388
UL36	1320	39	7.117	8.6346	0.0033	**0.01729**	--
UL37 Reg1	267	39	7.111	0	1	1	
UL37 Reg2	579	39	9.333	1.61734	0.20346	0.34205	
UL38	711	40	7.243	0.28817	0.5914	0.77081	
UL42	270	41	5.41	0.11004	0.7401	0.92314	
UL43	855	41	5.998	1.07204	0.30049	0.44122	
*UL44 **	1107	40	3.254	0	1	1	
*UL45 Reg1 **	768	38	6.302	8.86751	0.0029	**0.01604**	M162, P192, V266, F349, R380
*UL45 Reg2 **	1470	38	5.368	5.33291	0.02093	0.05921	
*UL46 **	870	41	4.362	11.48303	0.0007	**0.00621**	R199
*UL47 **	2688	41	8.580	12.72283	0.00036	**0.00381**	A24, R98, E141, G145, R779, A936
*UL48 Reg1 **	1686	40	9.808	6.03024	0.01406	**0.04661**	--
*UL48 Reg2 **	1806	40	10.989	1.79394	0.18045	0.32706	
*UL48 Reg3 **	1530	40	12.649	0.05825	0.80929	0.99768	
*UL48 Reg4 **	819	40	8.940	7.04548	0.00795	**0.02974**	V2050, V2159
UL49	1458	41	3.619	0.50408	0.47771	0.63695	
*UL50 **	531	40	2.673	1.44676	0.22905	0.36224	
*UL51 **	258	39	2.136	0	1	1	
*UL52 **	1386	41	4.247	8.70193	0.21339	0.34864	
*UL53 **	795	41	3.030	5.81204	0.01592	**0.04990**	--
*UL54 Reg1 **	1587	40	4.318	4.41033	0.03572	0.08633	
*UL54 Reg2 **	1353	40	4.887	3.55904	0.05922	0.12052	
*UL55 Reg1 **	1191	41	8.583	8.35151	0.00385	**0.01778**	E2, Y78, R393
*UL55 Reg2 **	1158	41	6.144	5.52376	0.01876	0.05580	
*UL56 Reg1 **	1053	40	1.248	0.00048	0.98259	1	
*UL56 Reg2 **	1164	40	3.399	14.41411	0.00015	**0.00227**	--
*UL57 Reg1 **	2694	38	6.695	1.18292	0.27676	0.41694	
*UL57 Reg2 **	693	38	6.753	0	1	1	
*UL69 **	1161	40	4.480	14.00462	0.00018	**0.00227**	--
*UL70 **	2553	41	4.942	8.37205	0.00381	**0.01778**	A216, G294, R465
*UL71 **	513	38	6.051	4.43295	0.03525	0.08633	
*UL72 **	915	41	5.742	0	1	1	
UL74 Reg1	315	38	2.368	1.21376	0.27059	0.41300	
UL74 Reg2	456	38	3.547	1.82256	0.17701	0.32592	
*UL75 Reg1 **	1275	40	8.377	5.75065	0.016483	0.05032	-
*UL75 Reg2 **	747	40	6.859	4.95663	0.02599	0.07178	
*UL76 **	705	41	3.270	5.42536	0.01985	0.05755	
*UL77 **	1629	41	4.667	3.80386	0.05113	0.10984	
UL78	897	40	11.772	3.67904	0.05510	0.11621	
UL79	771	38	2.945	3.83088	0.05032	0.10984	
*UL80 * (includes UL80.5)*	1119	41	4.335	14.21565	0.00016	**0.00227**	--
UL82	1194	40	17.656	8.11051	0.00441	**0.01827**	R510
UL84	1185	41	6.443	4.41467	0.03563	0.08633	

UL85 *	897	41	3.850	6.87921	0.00872	**0.03075**	Q191, A266
UL86 *	4005	41	4.044	13.87409	0.0002	**0.00227**	K365, P741, A1273
UL87	2298	41	4.726	18.95116	1.34 x 10^{-5}	**0.00083**	R34, P114, D174, S893
UL88 *	1161	41	8.116	8.0825	0.00447	**0.01827**	A414
UL89	2013	38	3.439	1.13924	0.28581	0.42505	
UL92	603	40	2.383	1.01927	0.31269	0.45340	
UL93 *	1344	41	4.639	15.42173	8.60 x 10^{-5}	**0.00227**	T546
UL94 *	1020	41	6.641	0.92969	0.33494	0.47382	
UL95 *	1020	40	3.538	1.75543	0.18520	0.32980	
UL96	381	41	4.718	0	1	1	
UL97 *	1488	41	4.831	0.50737	0.47628	0.63695	
UL98 *	1539	41	3.561	4.01743	0.04503	0.10046	
UL100 *	1014	40	7.126	0.93832	0.33271	0.47382	
UL102 *	2007	40	5.765	1.62341	0.20262	0.34205	
UL103 *	720	39	5.694	1.49463	0.22150	0.35686	
UL104 *	1866	41	3.757	1.34781	0.24566	0.37996	
UL105 *	2409	41	3.734	0	1	1	
UL111A	333	31	6.209	0	1	1	
UL112	780	36	4.060	0	1	1	
UL114 *	741	41	3.802	11.36135	0.00075	**0.00621**	R221
UL115 *	678	40	5.400	1.58848	0.20754	0.34393	
UL116	324	39	5.408	0	1	1	
UL117	825	41	4.864	0	1	1	
UL119	663	34	6.596	11.89177	0.00056	**0.00545**	A118, Y247
UL120	435	40	7.693	17.00397	3.73 x 10^{-5}	**0.00144**	P109, F171
UL122	1131	31	4.571	0.00074	0.97833	1	
UL123 Reg2	1002	29	7.573	9.60443	0.00194	**0.01251**	--
UL130	501	35	6.833	1.73601	0.18765	0.32980	
UL131A	315	30	6.696	4.81542	0.02821	0.07609	
UL132	330	40	8.142	0.67051	0.41287	0.56345	
UL141	705	38	7.551	4.72326	0.02976	0.078451	
UL144	255	38	6.708	0.85856	0.35414	0.49494	
UL145	294	39	5.857	0.03287	0.85613	1	
UL147	318	37	8.335	8.2903	0.00399	**0.01778**	--
UL148	504	38	10.732	3.21073	0.07316	0.14631	
US1	345	39	5.829	0	1	1	
US2	408	30	13.964	1.43417	0.23108	0.36224	
US3	366	31	11.921	0.04851	0.82567	0.99768	
US11	390	37	9.899	9.21816	0.0024	**0.01407**	--
US12	684	39	12.402	0	1	1	
US17	636	40	8.888	2.5403	0.11097	0.21819	
US18	579	40	11.522	1.98322	0.15905	0.30246	
US19	408	30	10.294	0.6839	0.40825	0.56345	
US20	699	41	9.856	0.02413	0.87656	1	
US21	636	41	8.900	0	1	1	
US22	1458	41	7.331	4.11933	0.04239	0.09643	
US23 Reg1	345	41	6.142	0	1	1	
US23 Reg2	1149	41	6.463	9.19608	0.00243	**0.01407**	T266, V280
US24	1395	41	5.749	6.87375	0.00875	**0.03075**	--
US26	1497	40	7.454	2.23462	0.13495	0.26090	
US28 Reg1	267	41	4.261	7.5144	0.00612	**0.02367**	--
US28 Reg2	474	41	4.946	9.9166	0.00164	**0.01118**	--
US29	741	41	4.58	15.1414	9.98 x 10^{-5}	**0.00227**	--
US30	498	30	9.586	0.43235	0.51084	0.67338	
US32	351	41	15.875	1.64434	0.19973	0.34205	

Notes: * Core genes; [a] Branch length is defined as number of nucleotide substitutions per codon. [b] $2\Delta lnL$: twice the difference of the natural logs of the maximum likelihood of the models being compared. [c] Positions refer to proteins of the Merlin strain (NC_006273).
