# Supplementary material for: Past and ongoing adaptation of human cytomegalovirus to its host
Source: PLoS Pathog. 2020 May 8;16(5):e1008476. doi: 10.1371/journal.ppat.1008476 (PMC7239485; doi:10.1371/journal.ppat.1008476)
Supplement: S4 Table — (PDF) [file ppat.1008476.s011.pdf]

S4 Table. List of primers.

Primer name	Sequence (5' to 3')
BAC mutagenesis [a]	
**vUL70 G294L Fw**	CGCTGACGCGGCGCCCGAAGCGCAGTGCACGCGCGAACCG**CTT**TTTCTGGCCGCCGGCTTTCAGGATGACGACGATAAGTAGGG
**vUL70 G294L Rv**	TGACGTCCAGCAGCTCGGTGGAAAGCCGGCGGCCCAGAAA**AAG**CGGTTCGCGCGTGCACTGCGCAACCAATTAACCAATTCTGATAG
**vUL70 G294A Fw**	CGCTGACGCGGCGCCCGAAGCGCAGTGCACGCGCGAACCG**GCA**TTTCTGGCCGCCGGCTTTCAGGATGACGACGATAAGTAGGG-3'
**vUL70 G294A Rv**	TGACGTCCAGCAGCTCGGTGGAAAGCCGGCGGCCCAGAAA**TGC**CGGTTCGCGCGTGCACTGCGCAACCAATTAACCAATTCTGATTAG
**vUL70 R465K Fw**	CGAAACCTGGCATCGCAGCCTCTTTCCGCGCGACCTGCTC**AAA**CACGTACCCGACAGCCGTTTAGGATGACGACGATAAGTAGGG
**vUL70 R465K Rv**	CGGTCAGGGCCTCGTCCGAGAAACGGCTGTCGGGTACGTG**TTT**GAGCAGGTCGCGCGGAAAGACAACCAATTAACCAATTCTGATTAG
**vUL70 R465A Fw**	CGAAACCTGGCATCGCAGCCTCTTTCCGCGCGACCTGCTC**GCA**CACGTACCCGACAGCCGTTTAGGATGACGACGATAAGTAGGG
**vUL70 R465A Rv**	CGGTCAGGGCCTCGTCCGAGAAACGGCTGTCGGGTACGTG**TGC**GAGCAGGTCGCGCGGAAAGACAACCAATTAACCAATTCTGATTAG
**UL70_Seq Fw**	ACACGGTCTGCGTGCACTAC
**UL70_Seq Rv**	AACTGTGTCTCGGGGCTGGT
gtA and gtB mutagenesis [b]	
**gtA-flag20-Fw**	CAGCTGGGCGTGACA**GATTACAAGGATGACGACGATAAG**AAGGTGTGCCAGCAT
**gtA-flag20-Rv**	ATGCTGGCACACCTT**CTTATCGTCGTCATCCTTGTAATC**TGTCACGCCCAGCTG
**gtB-flag20-Fw**	TGTATTGGCAAGACA**GATTACAAGGATGACGACGATAAG**AAGGTGTGCCAGCAT
**gtB-flag20-Rv**	ATGCTGGCACACCTT**CTTATCGTCGTCATCCTTGTAATC**TGTCTTGCCAATACA
**pCMV6-Seq Fw**	GAATTCGTCGACTGGATCCG
**pCMV6-Seq Rv**	CAGATCCTCTTCTGAGATGAG
Head-to-head in vitro competition [c]	
**UL70-294_ngs Fw**	TCGTCGGCAGCGTCAGATGTGTATAAGAGACAGGTCTGCGTGCACTACGTCTAC
**UL70-294_ngs Rv**	GTCTCGTGGGCTCGGAGATGTGTATAAGAGACAGTTCGTCGAGACGCAGTAGGTG
**UL70-465_ngs Fw**	TCGTCGGCAGCGTCAGATGTGTATAAGAGACAGGCGTACACCTGCCCAATGACCA
**UL70-465_ngs Rv**	GTCTCGTGGGCTCGGAGATGTGTATAAGAGACAGGACTTCGTGTCGCGTGTAGTAG

Notes:

[a] Primers contain sequences of homology upstream and downstream the sites to be mutated, the mutation (bold), and sequences homologous to the pEPkan-S template plasmid (underlined) (Addgene plasmid # 41017; http://n2t.net/addgene:41017; RRID:Addgene_41017) [1].

[b] Primers contain sequences of homology upstream and downstream the SP cleavage site and the DDK-tag sequence to be inserted (bolded).

[c] Sequences homologous to the UL70 sequence are underlined.

1. Tischer BK, von Einem J, Kaufer B, Osterrieder N. (2006) Two-step red-mediated recombination for versatile high-efficiency markerless DNA manipulation in escherichia coli. Biotechniques 40(2): 191-197.
