# Supplementary material for: Past and ongoing adaptation of human cytomegalovirus to its host
Source: PLoS Pathog. 2020 May 8;16(5):e1008476. doi: 10.1371/journal.ppat.1008476 (PMC7239485; doi:10.1371/journal.ppat.1008476)
Supplement: S5 Table — (PDF) [file ppat.1008476.s012.pdf]

S5 Table. Focus expansion assay (FEA).

TB40-BAC4 Strains	IEA POSITIVE FOCI / INFECTED CELL DILUTION
HFFs	
**Wild-type**	2 in 10^1 dilution
**vUL70 G294L**	6 in 10^1 dilution
**vUL70 R465K**	2 in 10^1 dilution
**vUL70 dm**	1 in 10^1 dilution
**vUL70 G294A**	5 in 10^1 dilution
**vUL70 R465A**	2 in 10^1 dilution
ARPE-19 cells	
**Wild-type**	7 in 10^3 dilution
**vUL70 G294L**	3 in 10^1 dilution
**vUL70 R465K**	3 in 10^3 dilution
**vUL70 dm**	1 in 10^2 dilution
**vUL70 G294A**	9 in 10^3 dilution
**vUL70 R465A**	6 in 10^3 dilution

Note: Serial dilutions of HFFs infected by the indicated viruses were co-cultured with an excess of uninfected HFF or ARPE-19 cells for 5 days. Monolayers were then fixed, and infected cells were traced by staining with antibodies against HCMV immediate early antigen (IEA), followed by immunoperoxidase assay. Infectious foci, defined as clusters of at least three infected cells, were counted in the indicated dilution.
