# Supplementary material for: Past and ongoing adaptation of human cytomegalovirus to its host
Source: PLoS Pathog. 2020 May 8;16(5):e1008476. doi: 10.1371/journal.ppat.1008476 (PMC7239485; doi:10.1371/journal.ppat.1008476)
Supplement: S6 Table — (PDF) [file ppat.1008476.s013.pdf]

S6 Table . List of HCMV strains used for HCMV ancestral outgroup reconstruction.

Strain Name	Accession ID	Sample Type	Sequence Length	Year	Country
JER4053	KR534207	Amniotic fluid	235126	2009	Israel
UKNEQAS2	KT634296	Amniotic fluid	234873	2013	Australia
CZ/1/2013	KP745691	Blood	235139	2013	Czech Republic
HANRTR1A	KY490073	Blood	235221	2012	Germany
JHC	HQ380895	Blood	235476	2003	South Korea
UK/Lon1/Blood/2013	KT726947	Blood	235143	2013	United Kingdom
2CEN5	KJ361947	Bronchoalveolar lavage	235567	2009	Germany
HAN21	KJ361951	Bronchoalveolar lavage	235834	2006	Germany
HAN39	KJ361957	Bronchoalveolar lavage	235056	2007	Germany
DB	KT959235	Cervical secretion	235512	2009	France
VR1814	GU179289	Cervical secretion	235233	1996	Italy
BE/33/2010	KP745661	Nasopharyngeal aspirate	235605	2010	Belgium
BE/40/2011	KP745722	Nasopharyngeal aspirate	235716	2011	Belgium
BE/45/2011	KP745633	Nasopharyngeal aspirate	235352	2011	Belgium
BE/21/2010	KC519322	Urine	235722	2010	Belgium
Merlin	NC_006273	Urine	235646	1999	United Kingdom
NANU	KU550090	Urine	235634	2013	France
