# Supplementary material for: Past and ongoing adaptation of human cytomegalovirus to its host
Source: PLoS Pathog. 2020 May 8;16(5):e1008476. doi: 10.1371/journal.ppat.1008476 (PMC7239485; doi:10.1371/journal.ppat.1008476)
Supplement: S7 Table — (PDF) [file ppat.1008476.s014.pdf]

S7 Table. List of HCMV strains used for gammaMap analyses**.**

Strain Name (additional information)	Accession ID	Sequence Length	Isolation Year	Country
Amniotic fluid				
AF1	GU179291	235937	2003	Italy
JER1070	KR534199	235492	2010	Israel
JER2002	KR534201	235339	2011	Israel
JER2282	KR534202	234549	2012	Israel
JER3230	KR534203	235857	2011	Israel
JER3855	KR534204	234804	2005	Israel
JER4035	KR534205	235314	2009	Israel
JER4041	KR534206	234917	2005	Israel
JER4053	KR534207	235126	2009	Israel
JER4559	KR534208	235673	2009	Israel
JER4755	KR534209	235266	2012	Israel
JER5268	KR534210	235445	2012	Israel
JER5409	KR534211	235943	2012	Israel
JER5550	KR534212	235160	2012	Israel
JER5695	KR534213	235797	2012	Israel
NAN1LA	KU550087	235062	2011	France
NAN2LA	KU550088	234396	2013	France
NAN4LA	KU550089	237120	2013	France
PAV1	KJ361959	235815	2005	Italy
PAV11	KJ361965	236310	-	Italy
PAV12	KJ361966	235616	-	Italy
PAV16	KJ872539	236240	2009	Italy
PAV18	KJ872540	234739	2008	Italy
PAV20	KJ872541	236293	2013	Italy
PAV21	KJ872542	235901	2013	Italy
PAV23	KJ361967	235700	2012	Italy
PAV24	KJ361968	235361	2012	Italy
PAV25	KJ361969	235902	2013	Italy
PAV26	KJ361970	236180	2013	Italy
PAV4	KJ361960	235272	2006	Italy
PAV5	KJ361961	235485	2006	Italy
PAV6	KJ361962	235432	2007	Italy
PAV7	KJ361963	235142	2007	Italy
PAV8	KJ361964	235432	2007	Italy
PRA6	KY490068	235717	2015	Czech Republic
UKNEQAS2	KT634296	234873	2013	Australia
Blood/plasma				
HANRTR10 (blood of kidney transplant recipient)	KY490078	234360	2010	Germany
HANRTR1A (blood of kidney transplant recipient)	KY490073	235221	2012	Germany
HANRTR1B (blood of kidney transplant recipient)	KY490074	235385	2013	Germany
HANRTR2 (blood of kidney transplant recipient)	KY123650	235472	2012	Germany
HANRTR4 (plasma of kidney transplant recipient)	KY123651	235329	2015	Germany
HANRTR8 (blood of kidney transplant recipient)	KY490076	235791	2013	Germany
HANSCTR11A (blood of stem cell transplant recipient)	KY490085	235632	2010	Germany
HANSCTR11B (blood of stem cell transplant recipient)	KY490086	234962	2010	Germany
HANSCTR12 (blood of stem cell transplant recipient)	KY490087	235848	2010	Germany
HANSCTR13 (blood of stem cell transplant recipient)	KY490088	235403	2011	Germany
HANSCTR1A (blood of stem cell transplant recipient)	KY490079	235579	2014	Germany
HANSCTR2	KY490081	235843	2015	Germany

(blood of stem cell transplant recipient)				
HANSCTR4<br>(blood of stem cell transplant recipient)	KY123653	235510	2011	Germany
HANSCTR8<br>(blood of stem cell transplant recipient)	KY490082	235058	2014	Germany
HANSCTR9<br>(blood of stem cell transplant recipient)	KY490083	235153	2016	Germany
JHC<br>(blood of bone marrow transplant recipient)	HQ380895	235476	2003	South Korea
PAV31<br>(plasma of transplant recipient)	KY490061	235221	-	Italy
PAV32<br>(plasma of transplant recipient)	KY490062	234316	-	Italy
UK/Lon1/Blood/2013<br>(immunocompromised patient)	KT726947	235143	2013	United Kingdom
Urine				
3301<br>(congenitally infected infant)	GQ466044	235703	2001	United Kingdom
BE/21/2010<br>(pulmonary transplant recipient)	KC519322	235722	2010	Belgium
HANChild1<br>(congenitally infected infant)	KY490071	235397	2013	Germany
HANChild2&3<br>(congenitally infected infant)	KY490072	235913	2013	Germany
NANU<br>(congenitally infected infant)	KU550090	235634	2013	France
PRA1<br>(congenitally infected infant)	KY490063	235826	2006	Czech Republic
PRA2<br>(congenitally infected infant)	KY490064	234791	2009	Czech Republic
PRA3<br>(congenitally infected infant)	KY490065	235442	2009	Czech Republic
PRA4<br>(congenitally infected infant)	KY490066	235513	2009	Czech Republic
PRA5<br>(congenitally infected infant)	KY490067	234989	2009	Czech Republic
PRA7<br>(congenitally infected infant)	KY490069	236373	2010	Czech Republic
PRA8<br>(congenitally infected infant)	KY490070	234832	2012	Czech Republic
U11<br>(congenitally infected infant)	GU179290	234732	2003	United Kingdom
U8<br>(congenitally infected infant)	GU179288	235709	2003	Italy
UK/Lon6/Urine/2011<br>(immunocompetent subject)	KT726949	235199	2011	United Kingdom
UK/Lon7/Urine/2011<br>(immunocompetent subject)	KT726950	235743	2011	United Kingdom
UK/Lon8/Urine/2012<br>(immunocompetent subject)	KT726951	235801	2012	United Kingdom
